# Camel milk fat-based human milk fat substitute

**DOI:** 10.3389/fnut.2026.1917713

**Published:** 2026-09-04

**Authors:** Ibrahim A. bakry, Wei Wei, Ning Li, Mohamed Abouzid, Xingguo Wang, Hazem Golshany, Abdelaziz Elbarbary, Alaa E. Nafea, Amal Gohary

**Affiliations:** 1 College of Food Science and Technology, Henan Agriculture University, Zhengzhou, Henan Province, China; 2 Department of Food and Dairy Science, Faculty of Technology and Development, Zagazig University, Zagazig, Egypt; 3 Collaborative Innovation Centre of Food Safety and Quality Control in Jiangsu Province, School of Food Science and Technology, Jiangnan University, Wuxi, China; 4 Department of Physical Pharmacy and Pharmacokinetics, Faculty of Pharmacy, Poznan University of Medical Sciences, Poznan, Poland; 5 Food Science Department, Faculty of Agriculture, Cairo University, Giza, Egypt

**Keywords:** camel milk, human milk, infant formula, palmitic acid, polyunsaturated fatty acids, similarity index

## Abstract

Human milk is the gold standard for infant nutrition. This study aimed to develop a camel milk fat-based human milk lipid mimetic whose fatty acid (FA) composition, structural properties, and *in vitro* digestibility more closely resemble those of human milk fat than conventional vegetable oil-based formulas. Camel milk (CM) fat was physically blended with sunflower, rapeseed, palm kernel, fish oils, and 1,3-dioleoyl-2-palmitoylglycerol using a computational optimization model to approximate the total and *sn-2* FA profiles of Egyptian human milk. The resulting formulation, together with three commercial formulas and human milk, was compared for total and *sn-2* FAs by gas chromatography, similarity indices, FTIR, DSC characteristics, and dynamic *in vitro* gastrointestinal digestion, including lipolysis degree and free FA release. IFCM contained 29 detectable fatty acids, matching human milk and exceeding commercial formulas ( ≤ 19), and reduced fatty acid mismatches with human milk to 11 vs. 12–19 in commercial products. Total saturated fatty acids reached 50.99%, and sn-2 palmitic acid 34.76%, closer to human milk than in commercial formulas. Intestinal lipolysis at 120 min was 66.47% for IFCM vs. 69.93% for human milk and 60.28%−65.43% for commercial formulas, while FTIR and DSC profiles of IFCM aligned more closely with human milk than with vegetable oil-based formulas. Combining CM fat with selected oils resulted in an infant formula with improved compositional and structural resemblance to human milk fat, as well as enhanced *in vitro* lipid digestion, compared to conventional formulas. IFCM represents a promising candidate human milk fat mimetic, warranting *in vivo* evaluation of its effects on infant growth, mineral absorption, and metabolic outcomes.

## 1 Introduction

Breastfeeding has numerous health benefits for both mothers and infants. It has been associated with a decreased risk of breast and ovarian cancers in mothers (1, 2). On the other hand, breastfed infants may have a reduced risk of obesity, diabetes, certain cancers, and cardiovascular diseases later in life (3). Additionally, fat accounts for approximately 50% of the energy intake of young infants, corresponding to around 25 grams per day during the first 6 months (4). Human milk fat absorbs vitamins and minerals, providing a source of energy. It also significantly affects infants' early neural development and physiological functions.

Unfortunately, only 44% of infants aged 0–6 months were exclusively breastfed (5). In this case, infant formula (IF) is an alternative to human milk (6). Thus, IF is the second-largest global food source after breast milk. Accordingly, researchers strive to optimize the composition and distribution of fatty acids (FAs) in IF to promote optimal infant growth and development. Understanding the relationships among the components of fats or oils, diet, and health is now recognized as a key concept for promoting a better lifestyle, preventing disease, and enhancing overall wellbeing. The multifactorial variability of human milk FA composition driven by maternal diet, genetics, lactation stage, and other factors necessitates careful consideration in infant formula design. Moreover, FA composition and positional distribution in IFs are fundamental to simulating human milk. Thus, replicating the exact composition of human milk fat in IF is challenging, given the significant variability in human milk FA composition (4).

Historically, human milk fat substitutes have been categorized into four main groups. (1) Early attempts: In the past, when human milk was unavailable or insufficient, various mammalian milks were used as substitutes. Cow milk was the most commonly used; however, its fat composition differs significantly from human milk. On average, cow milk contains 70% saturated fatty acids (SFAs), 25% monounsaturated fatty acids (MUFAs), and 5% polyunsaturated fatty acids (PUFAs) (7). As a result, milk fat has been criticized for its unfavorable FA profile; (2) Vegetable oil-based formulas: In the early 20th century, scientists began experimenting with vegetable oils as alternatives to mammalian milk fats. However, these formulas were often deficient in essential FA and fat-soluble vitamins in human milk. To compensate, vegetable oils were sometimes blended with mammalian milk fats to improve nutritional content; (3) Structured lipid-based substitutes: advances in enzymatic and chemical modification techniques enabled the production of structured lipids that more closely mimic the FA composition and positional distribution of human milk triacylglycerols (TAGs), particularly the high content of palmitic acid at the *sn*-2 position; (4) Over time, more recent developments in human milk fat substitutes have focused on creating IF that closely resemble the FA composition of human milk. These blends may include a combination of vegetable oils, structured lipids, and other specialized ingredients that better mimic the nutritional properties of human milk fat.

Despite these efforts, challenges remain in current IF products. These challenges include differences in the composition and distribution of FAs, lower proportions of polar lipids, and variations in milk fat globule membrane size (8). Additionally, cow milk allergy is one of the most common food allergies in infants and children, accounting for approximately 2%−3% (9). The prevalence of cow milk allergy in China ranges from 0.83 to 3.5% in various cities (10). These factors can impact the nutritional profile and functionality of the fats used in IFs, potentially affecting their similarity to human milk.

Camel milk (CM) fat recently garnered increasing attention due to its potential health benefits (11). At the same time, the FA profile of CM is closer to that of human milk, featuring a higher proportion of unsaturated FA and a more favorable distribution of palmitic acid at the *sn-2* position of TAG (12), which is important for efficient fat and calcium absorption in infants (13). CM has shown promise as a possible alternative to cow milk in IF (14, 15). Previous camel milk-based formula studies have largely emphasized CM as an alternative milk source or have focused on limited compositional properties. However, fewer studies have systematically optimized CM fat with selected vegetable and marine oils to more closely approximate both total and positional FA profiles of human milk. In addition, most earlier reports have not integrated compositional analysis with structural characterization and dynamic *in vitro* digestion. Therefore, the present study is novel in combining formulation optimization, total and *sn-2* FA profiling, FTIR and DSC characterization, and simulated gastrointestinal digestion to assess camel milk fat as a human milk fat mimetic.

## 2 Materials and methods

### 2.1 Chemicals and standards

Rabbit gastric extract (RGE-15U, lipase activity ≥15 U/mg) was purchased from Lipolytech (Marseille, France). Porcine bile (S31320) was obtained from Yuan Ye Bio-Technology Co., Ltd (Shanghai, China). Rapeseed, sunflower, fish, and palm kernel oil were purchased online from the Tmall supermarket. 1,3-Dioleoyl-2-palmitoyl triglyceride (O/P/O) was obtained from BeiJia Bio-Technology Co., Ltd. (Zhejiang, China). Porcine pancreatic lipase (type II) and silica gel board (20 mm × 20 mm) were purchased from Sigma-Aldrich Chemical Co. Ltd. (St. Louis, MO, United States). Analytical-grade ammonium hydroxide, ethanol, diethyl ether, petroleum ether, methanol, ammonium formate, potassium hydroxide, and anhydrous sodium sulfate were acquired from Sinopharm Chemical Reagent Co., Ltd. (Shanghai, China). HPLC-grade ethanol, acetonitrile, isopropanol, and n-hexane were purchased from Tedia Company, Inc. (Fairfield, OH, USA). We obtained a standard mixture containing 37 FA methyl esters (C4–C24).

### 2.2 Human milk and infant formula samples

Twenty-five samples of human milk were collected during the mature stage from healthy Egyptian women aged 25–35 years with full-term pregnancies who had not taken any medications before milk collection. The collection was conducted at Zagazig University Hospital. The study protocol was approved by the Medical Ethics Committee of Zagazig University Hospital (ZU-IRB no.10326/5-2023). The samples were collected between 9:00 and 11:00 AM, using an electric breast pump, and divided into two parts: one for lipid analysis and the other for the digestion experiment. The samples were transported in an ice box and transferred to Jiangnan University in China. Fresh CM was obtained from a local producer in China. The characteristics of the camels and their corresponding milk samples are summarized in Supplementary Table 1. The milk was collected and mixed under strict hygienic conditions into a sterile milking can. The samples were transported by car to the laboratory at Jiangnan University, China, within 10 h in insulated iceboxes, maintaining temperatures of −80 to prevent changes in the composition of CM fat. Additionally, three commercial plant oil-based powdered infant formulas (IF1, IF2, and IF3) were purchased from prominent supermarkets in Zagazig, Egypt. According to the product labels, IF1 contained soybean oil, sunflower oil, and coconut oil; IF2 contained high-oleic sunflower oil, coconut oil, and soybean oil; and IF3 contained high-oleic sunflower oil and soybean oil. These formulas were intended for infants aged 6–12 months and were used as commercial comparator formulas for comparison with the experimental IFCM. The IFs were dissolved in water at approximately 37 °C to obtain a 10% (w/v) solution.

### 2.3 Preparation of a novel infant formula by physical blending

Based on the FA composition and distribution of Egyptian human milk (Table 1) and by the guidelines established by the United States Department of Agriculture (DHA at 0.2%−0.5% of total FA, with ARA equal to or exceeding DHA, to mimic human milk fat) (16). CM fat was blended with specific proportions of sunflower oil (SO), fish oil (FO), rapeseed oil (RO), 1,3-dioleoyl-2-palmitoylglycerol (O/P/O), and palm kernel oil (PO). The blending ratios were determined based on the FA composition and distribution of each oil, as shown in Supplementary Tables 2, 3. Supplementary Table 4 presents the theoretical and actual lipid compositions and contents of the resulting oil blends. A weighted arithmetic mean was applied for each FA to identify the optimal proportions of each oil. This approach assessed the contribution of each oil to the target FA profile based on its weight relative to the other components in the mixture. The total and *sn*-2 positional FA profiles of blended oils were subsequently calculated using a previously validated physical blending model (17).


$$\begin{array}{l} \%FA=(FA_{\text{CM}}\;\times \;X_{\text{CM}}\;+\;FA_{\text{SO}}\;\times \;X_{\text{SO}}\;+\;FA_{\text{FO}}\;\times \;X_{\text{FO}}\\ +\;FA_{\text{RO}}\;\times \;X_{\text{RO}}+\;FA_{\text{O/P/O}}\;\times \;X_{\text{O/P/O}}\;+\;FA_{\text{PO}}\;\times \;X_{\text{PO}}) \end{array}$$ (1)

$$\begin{array}{l} \%sn-2FA\;=\;(sn-2FA_{\text{CM}}\;\times \;X_{\text{CM}}\;+\;sn-2FA_{\text{SO}}\;\times \;X_{\text{SO}}\\ +\;sn-2FA_{\text{FO}}\;\times \;X_{\text{FO}}\;+\;sn-2FA_{\text{RO}}\;\times \;X_{\text{RO}}\;+\;sn-2FA_{\text{O/P/O}}\\ \times \;X_{\text{O/P/O}}\;+\;sn-2FA_{\text{PO}}\;\times \;X_{\text{PO}}) \end{array}$$ (2)

Where % FA is the amount of the corresponding FA in the blend (mol%), FA_CM_ is the amount of the FA in CM (mol%), X_CM_ is the fraction of CM in the mixture, FA_SO_ is the amount of the FA in SO (mol%), X_SO_ is the fraction of SO in the blend, and so forth. % *sn*−*2* FA followed the same explanation.

**Table 1:** Total fatty acid compositions of human milk and infant formula^**a**^.

FA	Human milk	IFCM	IF1	IF2	IF3
6:0	0.19 ± 0.22	0.15 ± 0.01	0.21 ± 0.04	0.22 ± 0.02	0.43 ± 0.06
8:0	0.32 ± 0.26^c^	0.45 ± 0.01^c^	1.94 ± 0.09^a^	1.88 ± 0.08^a^	1.16 ± 0.05^b^
10:0	1.57 ± 0.84	0.48 ± 0.01	1.91 ± 0.13	1.85 ± 0.08	1.12 ± 0.03
12:0	7.99 ± 2.63	6.09 ± 0.03	5.79 ± 0.29	5.73 ± 0.10	9.83 ± 0.12
13:0	0.04 ± 0.03	0.04 ± 0.01	0.02 ± 0.00	0.02 ± 0.00	ND
14:0	8.82 ± 2.55^a^	7.33 ± 0.00^ab^	4.33 ± 0.05^b^	4.27 ± 0.07^b^	4.65 ± 0.08^b^
14:01	0.12 ± 0.04^b^	0.19 ± 0.01^a^	0.08 ± 0.09^b^	0.14 ± 0.08^ab^	ND
15:0	0.25 ± 0.05^b^	0.60 ± 0.01^a^	0.11 ± 0.12^c^	0.14 ± 0.04^c^	ND
15:01	0.05 ± 0.02^b^	0.13 ± 0.00^a^	0.01 ± 0.01^c^	ND	ND
16:0	24.11 ± 2.97^a^	24.65 ± 0.45^a^	17.53 ± 0.8^b^	16.59 ± 0.01^b^	19.49 ± 0.45^ab^
16:01	1.85 ± 0.60^a^	2.27 ± 0.05^a^	0.40 ± 0.01^b^	0.39 ± 0.01^b^	0.26 ± 0.08^b^
17:0	0.31 ± 0.08^a^	0.34 ± 0.05^a^	0.07 ± 0.01^b^	0.12 ± 0.08^b^	0.08 ± 0.00^b^
17:01	0.14 ± 0.04^a^	0.17 ± 0.01^a^	0.12 ± 0.01^ab^	0.11 ± 0.01^ab^	0.07 ± 0.01^b^
18:0	5.47 ± 1.70^b^	10.40 ± 0.16^a^	4.50 ± 0.00^b^	4.17 ± 0.21^b^	3.75 ± 0.06^b^
18:1 (*n*−9)	25.11 ± 2.71^b^	27.87 ± 0.14^b^	43.45 ± 0.35^a^	45.23 ± 0.32^a^	41.63 ± 0.51^a^
t18:2	0.15 ± 0.10^a^	0.20 ± 0.01^a^	0.04 ± 0.05^ab^	0.05 ± 0.02^ab^	ND
18:2	19.38 ± 3.96	15.62 ± 0.18	14.58 ± 0.60	14.48 ± 0.13	14.26 ± 0.08
18:3 *n*−6	0.19 ± 0.09^a^	0.05 ± 0.01^b^	0.16 ± 0.04^ab^	0.14 ± 0.00^ab^	ND
18:3 *n*−3	0.94 ± 0.38^d^	1.14 ± 0.01^cd^	2.39 ± 0.04^a^	2.12 ± 0.04^ab^	1.68 ± 0.03^bc^
20:0	0.14 ± 0.07^b^	0.34 ± 0.01^a^	0.34 ± 0.01^a^	0.33 ± 0.02^a^	0.32 ± 0.01^a^
20:01	0.31 ± 0.11^b^	0.14 ± 0.01^c^	0.45 ± 0.01^a^	0.47 ± 0.03^a^	ND
20:2 n−6	0.53 ± 0.11^a^	0.01 ± 0.01^c^	0.36 ± 0.01^b^	0.37 ± 0.02^b^	0.50 ± 0.00^ab^
20:4 n−6	0.54 ± 0.20^a^	0.24 ± 0.06^b^	ND	ND	ND
20:3 n−3	0.42 ± 0.13	0.05 ± 0.00	ND	0.13 ± 0.06	ND
20:05	0.11 ± 0.08^b^	0.44 ± 0.03^a^	0.09 ± 0.01^a^	0.38 ± 0.01^b^	ND
22:0	0.17 ± 0.13	0.15 ± 0.00	0.37 ± 0.01	0.16 ± 0.04	0.28 ± 0.01
22:01	0.12 ± 0.06^a^	0.07 ± 0.01^ab^	0.13 ± 0.01^a^	0.03 ± 0.05^b^	ND
24:01	0.05 ± 0.04^b^	0.04 ± 0.00^b^	0.48 ± 0.01^a^	0.48 ± 0.01^a^	0.51 ± 0.00^a^
22:6 *n*−3	0.49 ± 0.17^a^	0.47 ± 0.01^a^	0.17 ± 0.17^b^	0.04 ± 0.01^b^	ND
SFA	49.34 ± 5.71^a^	50.99 ± 0.38^a^	37.1 ± 0.99^b^	35.47 ± 0.38^b^	41.09 ± 0.46^b^
MUFA	27.75 ± 2.73^c^	30.80 ± 0.20^b^	45.1 ± 0.25^a^	46.84 ± 0.36^a^	42.46 ± 0.42^a^
PUFA	22.73 ± 4.55	18.20 ± 0.17	17.78 ± 0.72	17.71 ± 0.03	16.44 ± 0.11
LA/ALA	22.29 ± 5.34^a^	13.76 ± 0.25^b^	6.10 ± 0.36^b^	6.85 ± 0.18^b^	8.49 ± 0.10^b^

^a^Values are the mean±SD (n= 3); Values in the same row with different superscript letters (a, b, c, d, e) are significantly different among human milk and infant formulas at (*p* < 0.05).

IF, infant formula; FA, fatty acid; SFA, saturated fatty acid; MUFA, monounsaturated fatty acid; PUFA, polyunsaturated fatty acid; LA/ALA, linoleic acid/alpha-linolenic acid; ND, not detected. Numbers with the same alphabetical letter denote significant differences between these numbers.

IFCM was evaluated using a model developed by Al-Abdi et al. to ensure the quality of the final product (18). The model aimed to assess the extent to which the produced IF resembled human milk fat.


$$\begin{array}{l} ASI\;\left(fat\right)=\;\frac{1}{n}\;\left(ISI\;\left(fat1\right)\;+\;ISI\;\left(fat2\right)\;...ISI\;\left(fat\;n\right)\right) \end{array}$$ (3)

Where average similarity index (ASI) (fat) is the ASI and individual similarity indices (ISI) (fat1...fat n) are the individual similarity indices of the selected fat elements, and f and F are smaller values and the higher values, according to Bray and Curtis (19). To ensure an ideal blending ratio, we have also developed a Python program to optimize the entire blending process.


$$\begin{array}{l} Similarity\;index=\frac{2\sum_{i=1}^{n}minimum\;\left(Ai,\;Mi\right)}{\sum_{i=1}^{n}Ai+\sum_{i=1}^{n}Bi} \end{array}$$ (4)

Where *Ai* and *Bi* are the abundances of species *i* in sites A and B, respectively, *n* is the total number of species. Min (*Ai, Bi*) is the smaller abundance of species *i* found in both sites. We denote the smaller value as *f* and the higher value as *F*, and thus, each ISI can be presented as


$$\begin{array}{l} ISI\left(fat\right)=\frac{2\times f}{f+F} \end{array}$$ (5)

Finally, the IFCM was prepared by blending CM fat, which constituted 40% of the total fat in IF, with a combination of different oils. The proportions used were SO, FO, RO O/P/O, and PO at 5, 5, 15, 20, and 15%, respectively. This specific ratio was chosen to closely resemble the FA composition and distribution of human milk fat.

### 2.4 Producing infant formula

After collection, CM was immediately pasteurized at 70 °C for 5 min. The milk was then cooled down to 40 °C. The cream was separated from the milk using an Alfa Laval separator (Alfa Laval Separator Co., New Zealand). The aqueous phase consisted of skim milk, and the oil phase consisted of camel cream and selected oils. The oil phase was added to the aqueous phase, and the mixture was heated to 50 °C in a water bath. It was then homogenized with a high-speed Ultra-Turrax blender (T25, IKA, Germany) at 15,000 rpm for 2 min to form a crude emulsion.

**Figure 1:**
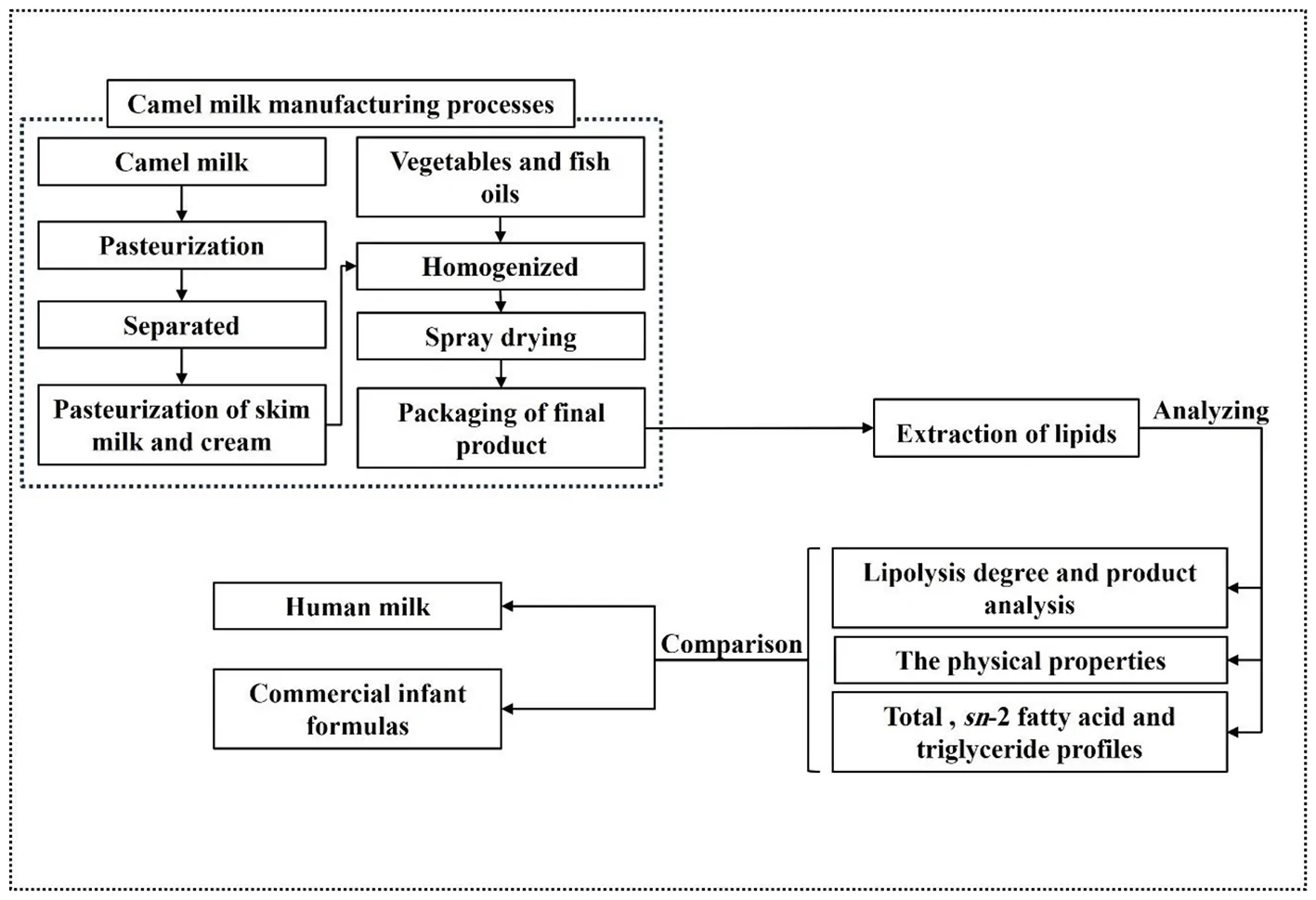
Technical roadmap for a camel milk-based lipid formulation.

The emulsion was transferred into a high-pressure homogenizer (HPH2000/4-SH5, GEA, Germany) and subjected to four cycles at 60/620 bar (first-stage pressure/second-stage pressure) to obtain IFCM. After homogenization, the next step was spray drying. The spray dryer used for this purpose was the BUCHI b-290 model, manufactured in Switzerland. It was equipped with a liquid nozzle capable of evaporating 1 liter of water per hour. The atomic compressor air intake was set at 40 kPa to operate the spray dryer. The drying air temperatures were closely monitored and controlled throughout the process. The inlet temperature of the drying air was maintained at 160 °C, while the outlet temperature was set at 70 °C. These controlled temperatures during spray drying help remove moisture from the mixture, resulting in the formation of IFCM powder. After the spray drying, the powders obtained were collected and placed in a vacuum-sealed aluminum bag for packaging (Figure 1).

### 2.5 *In vitro* digestion experiment

The dynamic *in vitro* simulated digestion model was conducted based on the methodology described in previous studies (20–22). The *in vitro* digestion involved simulating the stomach and small intestine using two 100-mL double-jacketed glass reactors for each. Before digestion, samples were equilibrated to 37 °C, and the pH was adjusted to the required starting value using 0.1 M HCl or NaOH.

Gastric digestion was conducted on 50 mL of milk in a 100 mL double-walled glass reactor (gastric phase) at 37 °C with vigorous shaking for 120 min. Simulated gastric fluid (SGF) containing RGE (dissolved in 19.2 U/mL gastric content) was introduced into the gastric phase using a peristaltic pump. After 30 min, partial gastric content transfers were allowed *via* a pump at rates determined from a previous study (22). Simulated intestinal fluid (SIF), pancreatic lipase enzymes (dissolved in SIF), and bile salts (also dissolved in SIF) were introduced into the intestinal phase using pumps. The pH was maintained at 6.5 by automatic titration with 0.1 M NaOH, and the volume of NaOH consumed was recorded to estimate free FA release. Samples were collected at 30, 60, 90, and 120 min, immediately frozen at −80 °C, and stored for subsequent lipid extraction and analysis.

### 2.6 Fat extraction

Lipids were extracted using the Röse-Gottlieb procedure based on the ISO-IDF method (23). In brief, 10 mL of each milk sample was mixed with 2.0 mL of ammonia solution (25%−28%) to extract the milk fat. The mixture was then heated to 65 °C for 15–20 min, cooled to room temperature, and supplemented with 10 mL of ethanol, 25 mL of ethyl ether, and 25 mL of petroleum ether. After thorough blending, the upper supernatant layer was carefully transferred into a fresh tube and left undisturbed for 1 h to facilitate separation. The 95% ethanol from the third extraction was discarded, and an additional 5 mL of 95% ethanol, 12.5 mL of ethyl ether, and 12.5 mL of petroleum ether were added to the remaining solution, followed by vigorous mixing. The ether solutions from the three extractions were combined, and the resulting fat extract was subsequently dried for further analysis.

### 2.7 Determination of total and *sn*-2 fatty acid composition

FA compositions of fat samples were analyzed using the standard procedure (24). A sample (10 mg) was dissolved in 700 μL hexane, and then 125 μL of KOH–CH3OH (2 molL^−1^) was added and mixed for 5 min, followed by 25 μL of a saturated solution of sodium methoxide. The top layer was collected after blending and drying over sodium sulfate, and analyzed by gas chromatography (GC Agilent 7820A). FA were detached with a capillary column (TRACE TR-FAME, 60 m × 0.25 mm × 0.25 μm, Thermo Fisher, Waltham, MA, United States). During analysis, chromatographic conditions were optimized. Nitrogen gas was used as the carrier gas at a flow rate of 1.2 mLmin^−1^, with an injector temperature of 250 °C, FID temperature of 250 °C, and a split rate of 1:100; detector gases were a mixture of 30 mLmin^−1^ hydrogen, 400 mLmin^−1^ air, and 25 mLmin^−1^ nitrogen. FA analysis was performed in temperature-gradient mode, starting at 60 °C min^−1^ and increasing by 5 °C min^−1^ to 175 °C (15 min), then by 2 °C min^−1^ to 220 °C (10 min).

Retention times of GC peaks were matched with those of a mixture of the FA.

The positional distribution of fat samples was determined by pancreatic lipase-catalyzed hydrolysis, as described by Luddy (25). Samples (0.1g) were mixed with 7 mL of Tris buffer (pH 7.5), 0.7 mL of 2.2% CaCl_2_, 1.75 mL of 0.05 % bile salts, and 30 mg of pancreatic lipase as a catalyst. The mixture was initially incubated on a shaking water bath at 40 °C for 3 min with vigorous blending (three times), then cooled to room temperature, and 2 mL of diethyl ether was added. The mixture was then centrifuged at 4,500 rpm for 5 min. The top layer was separated, and diethyl ether was evaporated under N2 to a final volume of 500 μL. Thin-layer chromatography plates were used to separate the 2-monoacylglycerol (2-MAG) using an established solvent system composed of diethyl ether/acetic acid/hexane (50:1:50, v/v/v) for 60 to 70 min. Bands corresponding to 2-MAG were located using UV light and scraped off for methylation. Finally, the positions of FA were determined using gas chromatography (GC).

### 2.8 Lipolysis degree and product analysis

The lipid compositions, including TAG, diacylglycerol (DAG), MAG, and FFA, of the lipolysis products during digestion were analyzed using HPLC equipped with a refractive index detector and a Sepax HP-Silica column (4.6 mm × 250 mm × 5 μm, Sigma-Aldrich Corp., Tokyo, Japan). The mobile phase consisted of n-hexane, isopropyl alcohol, and formic acid (15:1:0.003, v/v/v) at a flow rate of 1 mL/min. As previously described, the lipid samples were dissolved in n-hexane (20 mgmL^−1^) and injected into the column at 35 °C (20). The lipolysis degree (LD) was calculated using the following equation (21).


$$\begin{array}{l} LD\;\left(\%\right)=\frac{100\;\times \;\left[FFA\right]}{3\;\times \;\left[TAG\right]\;+\;2\;\times \;\left[DAG\right]\;+\;\left[MAG\right]\;+\;\left[FFA\right]} \end{array}$$ (6)

Where [FFA], [TAG], [DAG], and [MAG] are the concentrations (M) of FFA, TAG, DAG, and MAG, respectively.

Analyzing the release of FFAs involves studying the liberation of FFAs from samples during pancreatin's interaction with TAGs in SIF. During complete digestion, each TAG molecule produces at least one FFA, and the release of FFAs can indicate the extent and rate of digestion. A volume of 0.1 M NaOH was used to determine the amount of FFA released during intestinal digestion, according to the equation described by Li and McClements (17).


$$\begin{array}{l} FFA\;\left(\%\right)=100\times \left(\frac{V\;NaOH\;\times M\;NaOH\;\times \;M\;lipid}{W\;lipid\times 2}\right) \end{array}$$ (7)

Here, V_NaOH_ is the volume of NaOH consumed to neutralize the FFAs (mL), M _NaOH_ is the molarity of the NaOH (M), M_lipid_ is the average molecular weight of the sample lipids (gmol^−1^), and W_lipid_ is the total mass of lipid initially present in the digestion vessel (g).

### 2.9 Determine the physical properties of human milk and infant formulas.

An FT-IR analyzer (IS10, Thermo Nicolet Inc., United States) equipped with a diamond crystal cell (ATR) accessory was used in this chapter. The extracted fat samples were homogeneously spread onto the surface of the ATR crystal, and the FT-IR spectra were recorded from 400 to 4,000 cm^−1^ at a resolution of 4 cm^−1^ by 32 scans. After scanning each sample, the spectra were subtracted against the background air spectrum, and ATR crystal was cleaned with ethanol and hexane. The cleanliness of ATR crystal was verified. Measurements were repeated several times, and spectra were averaged.

Human milk and IFs were analyzed for fat melting and crystallization profiles using a DSC2000 by TA Instruments in Leatherhead, United Kingdom (26). Before analysis, calibration was done using dodecane, eicosane, and indium as reference standards. Five to ten milligrams of the fat extract were weighed in an aluminum pan using an empty pan as the reference. Then, samples were subjected to a temperature range from 25 to 80 °C at 50 °C min^−1^, maintained at 80 °C for 10 min, cooled to −55 °C at 10 °C min^−1^ and kept at −55 °C for 10 min, and finally heated to 80 °C at 5 °C min^−1^. Thermal Analysis Software (TA Instruments) was used to analyze thermographs.

### 2.10 Statistical analysis

The analyses of IF samples were performed in triplicate and human milk samples in duplicate; these replicates denote analytical (technical) replicates rather than additional biological samples. Human milk specimens were analyzed individually (*n* = 25 donors), not pooled. CM used to prepare the experimental formula was obtained from a single production batch and processed under the same conditions for all preparations. Each commercial formula was purchased as an individual retail product and analyzed from a single lot per product. For statistical comparisons, analytical replicate values were averaged to produce one mean value per biological sample (human milk donor) or per formula preparation; all inferential tests were performed on these sample-level means. Results are reported as mean ± standard deviation (SD). Between-group differences were assessed using the Newman–Keuls multiple-comparison test implemented in CoStat v6.45 (CoHort Software, United States) with significance set at *P* < 0.05. Multivariate analyses (PCA, VIP, hierarchical clustering heat map) were performed in MetaboAnalyst (version 5.0, https://www.metaboanalyst.ca/home.xhtml), using the sample-level means as input.

## 3 Results and discussion

### 3.1 Theoretical and actual value of camel milk fat with selected oils

Human milk fats are characterized by their unique composition and positional distribution of FA, which are crucial for infant nutrition (4). Formula milk formulations aim to replicate these properties, providing infants with optimal nutrition. Our previous study found that the composition and distribution of FA in commercial IFs in Egypt differed from those in human milk (27). Thus, CM fat is an attractive starting material for IF due to its similarity to human milk in terms of FA composition, particularly the higher proportion of palmitic acid (16:0) at the *sn–*2 position compared to other animal milk sources (11). However, to fully meet infant nutritional needs, it is necessary to provide CM with essential FAs, including the *n–*3 and *n–*6 FAs (12).

A common approach involves blending different vegetable oils with milk fats to closely mimic the composition of human breast milk. FA composition and distribution profile of the CM fat with selected oils are shown in Supplementary Tables 2, 3. Our study integrated CM fat with various oils, including PO, O/P/O, RO, SO, and FO, to create an IFCM. Our data revealed that CM and PO were crucial sources of SFA. At the same time, O/P/O was a significant source of MUFA, and both RO and SO contributed to PUFA levels.

Furthermore, FO provided essential FA such as *n–*3 and *n–*6 (Supplementary Table 3). The composition of FA at the *sn–*2 position in CM fat and selected oils is outlined in Supplementary Table 3. PO exhibited 38.32% of 12:0 at the *sn–*2 position, whereas CM fat and O/P/O oil contained 40.46 and 66.17% of 16:0, respectively. Conversely, rapeseed and sunflower oil had approximately 70.96 and 65.39% of 18:2, respectively. FO contributed various types of FA, including 20:5n–3 and 22:6n–3. The IFCM was developed by blending CM fat, RO, SO, FO, PO, and O/P/O oils based on an analysis of FA composition and distribution in human milk (Table 1).

### 3.2 Fatty acid composition of human milk and infant formula profile

**Figure 2:**
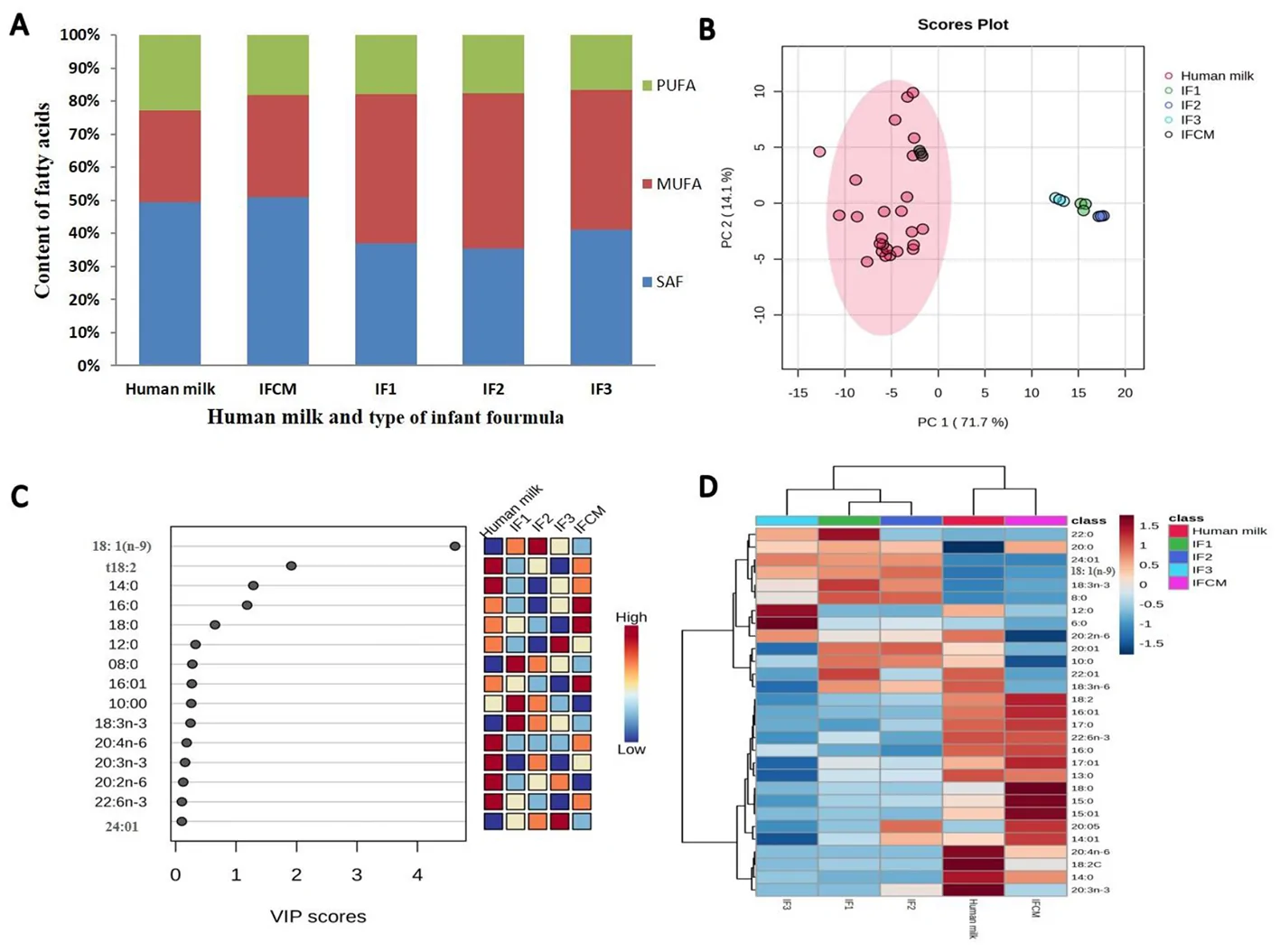
Total FAs content of human milk and infant formulas **(A)**, principal component analysis (PCA) **(B)**, variable importance in the projection (VIP, **C**), and hierarchical clustering heatmap **(D)**. IF, infant formula; IFCM, infant formula with camel milk; SFA, saturated fatty acids; MUFA, monounsaturated fatty acids; PUFA, polyunsaturated fatty acids.

The composition of total FA in human milk and all IF is shown in Table 1. Twenty-nine FAs were detected in human milk and IFCM, consisting of twelve SFAs, eight MUFAs, and nine PUFAs. In human milk and IFCM, SFAs and MUFAs were the primary types of FAs, followed by PUFAs. On the other hand, in commercial IFs, MUFAs and SFAs were the most prevalent FAs, followed by PUFAs (Figure 2A). This study also identified specific differences in the types of FAs observed between human milk and the different IFs. There were eleven significant differences between human milk and IFCM, fifteen between human milk and IF1, twelve between human milk and IF2, and nineteen between human milk and IF3. Consequently, the rationale behind choosing these oils as blend components was their high levels of specific FA, which played a crucial role in effectively aligning with the composition of human milk fat.

When comparing IFCM to commercial IFs, we observed 16, 15, and 8 significant differences in the types of FA detected, respectively. For example, (1) IFCM has lower levels of 8:0 compared to commercial IFs: [0.45 ± 0.01 vs. 1.94 ± 0.09, 1.88 ± 0.08, and 1.16 ± 0.05]; (2) higher levels of 14:01, 15:0 and 16:0 compared to IF1 and IF2; (3) higher levels of 17:0, 17:01 and 18:0 compared to commercial IFs; (4) lower levels of 20:01 and 20:2 n–6 compared to IF1 and IF2, and; (5) higher levels of SFAs compared to IFs [50.99 ± 0.38 vs. 37.1 ± 0.99, and 35.47 ± 0.38] (Table 1). The contrasting fat sources used in IFCM and commercial IFs may account for the differences in FA proportions observed between them. These differences likely reflect variations in the FA profiles of the different sources and formulations of IFs compared to human milk. Because the human milk reference samples were collected only from mature milk of Egyptian women, the present findings reflect similarity to this specific regional cohort and should not be generalized to all human milk populations. However, these findings support the model's reliability in determining the proportions of oil in the blend.

Based on the FA profile assessed in this study, the formula aligns with the selected compositional recommendations set by the European Commission and ESPGHAN, especially regarding ARA, DHA, and the LA/ALA ratio. However, this interpretation is limited to the FA composition evaluated in our study. Full regulatory compliance cannot be confirmed from the current data alone and would require additional assessment of nutrients, contaminants, microbiological quality, and other safety parameters in future research.

To explore the relationships between human milk and IFs in more detail, PCA, VIP, and hierarchical cluster heatmaps were performed using Metaboanalyst 6.0. The PCA score plot was used to examine differences in total FAs in human milk and all IFs (Figure 2B). It helps visualize how samples cluster or separate based on their FA characteristics. The first and second principal components accounted for 85.8% of the variance, indicating that the breast milk and commercial IF samples could be divided into two distinct groups. However, IFCM samples clustered in the same region of human milk, suggesting similarities in total FA °Composition.

Figure 2C displays the contribution of each variable to the classification. Based on the mean decrease, all essential FAs, except 18:1(*n–*9), were found to have low content. These results suggest that low-abundant FA may be more crucial in classifying human milk and IFs. A hierarchical clustering heat map was generated from the FA profiles of human milk and IFs to examine the distribution across the different FA groups. The cluster analysis results were similar to those obtained from PCA (Figure 2D). The heat map revealed the following FA distribution in the groups: (1) Human milk exhibited a relatively high content of 20:4 *n–*6, 18:2, 14:0, and 20:3 *n–*3 FAs. (2) IFCM showed a relatively high content of 18:0, 15:0, and 15:01 FAs. (3) IF1 had a relatively high content of 22:0 and 18:3 *n–*3 FAs. (4) IF3 exhibited the highest contents of 12:0 and 6:0 FA.

### 3.3 *Sn*-2 fatty acid composition of human milk and infant formula profiles

**Figure 3:**
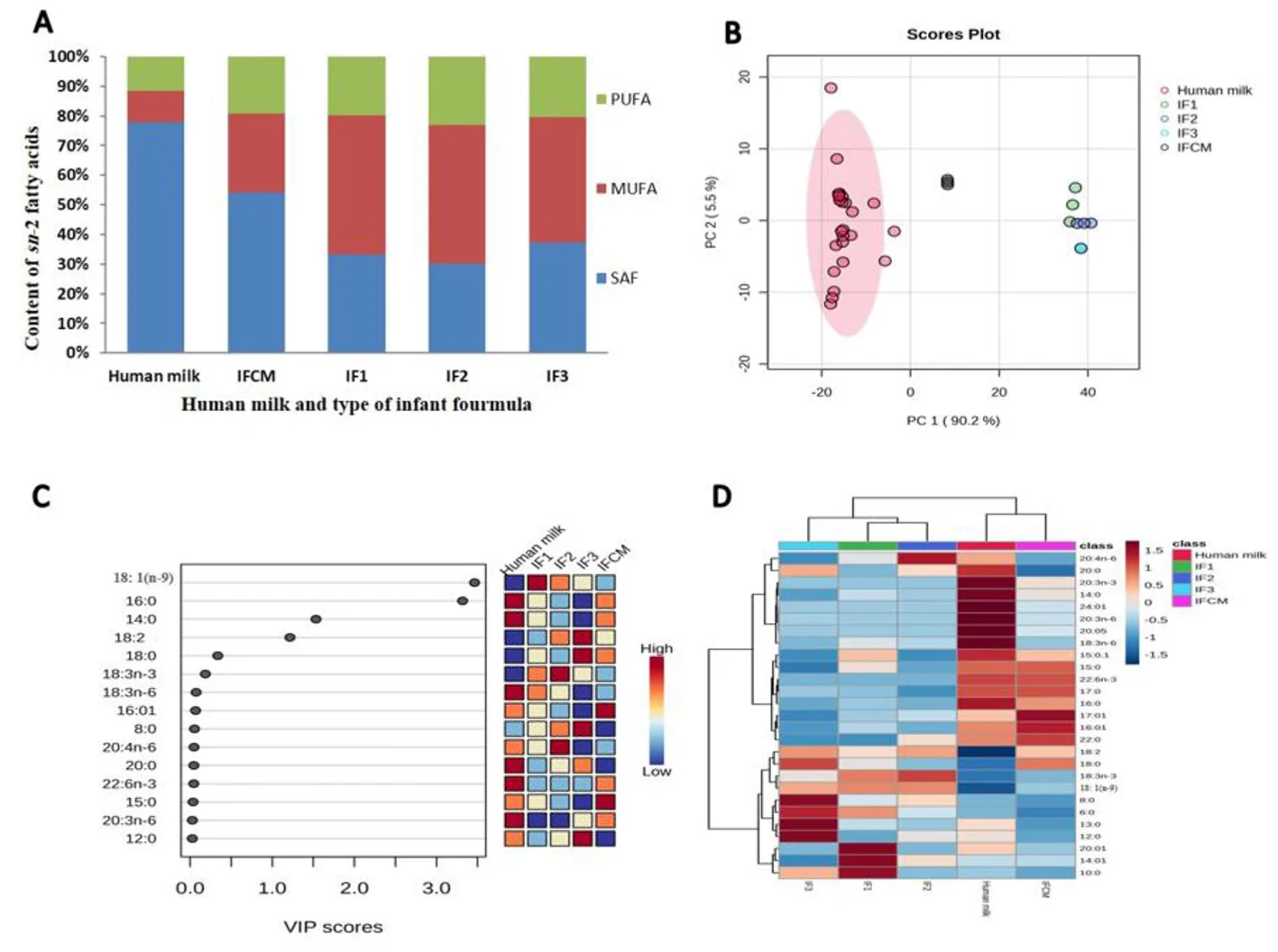
*sn*-2 FAs content of human milk and infant formulas **(A)**, principal component analysis (PCA) **(B)**, variable importance in the projection (VIP, **C**), and hierarchical clustering heatmap **(D)**. IF, infant formula; IFCM, infant formula with camel milk; SFA, saturated fatty acids; MUFA, monounsaturated fatty acids; PUFA, polyunsaturated fatty acids.

The composition of *sn–*2 FA in human milk and IFs is crucial for infant nutrition, particularly in fat absorption and calcium utilization. Table 2 presents the composition of *sn*−*2* FA in human milk and all formulas. We identified 28 *sn*−*2* FA in human milk and IFCM, while IF1, IF2, and IF3 had 21, 20, and 15 *sn*−*2* FA, respectively. Based on their degree of unsaturation, these *sn*−*2* FAs were categorized into three groups: SFAs, MUFAs, and PUFAs (Figure 3A). When comparing human milk to IFCM, a notable difference in *sn*−*2* SFA content was observed, with human milk having higher values (77.75 vs. 54.01%). However, when comparing IFCM to commercial formulas, the SFA content was higher in IFCM compared to commercial IFs.

**Table 2:** *sn*−2 fatty acids compositions of human milk and infant formula.

FA	Human milk	IFCM	IF1	IF2	IF3
6:0	0.10 ± 0.06^d^	0.02 ± 0.00^e^	0.36 ± 0.05^b^	0.19 ± 0.26^c^	0.48 ± 0.0^a^
8:0	0.12 ± 0.07^c^	0.01 ± 0.00^c^	0.39 ± 0.40^b^	0.58 ± 0.50^b^	1.25 ± 0.01^a^
10:0	0.29 ± 0.13^c^	0.11 ± 0.01^c^	1.54 ± 0.93^a^	0.22 ± 0.31^c^	0.83 ± 0.04^b^
12:0	9.36 ± 2.93^b^	5.95 ± 0.04^b^	6.08 ± 0.83^b^	9.11 ± 0.03^a^	15.38 ± 0.06^a^
13:0	0.51 ± 0.63	0.06 ± 0.00	0.32 ± 0.21	0.21 ± 0.17	1.10 ± 0.00
14:0	16.57 ± 4.52^a^	7.19 ± 0.07^b^	5.55 ± 0.34^b^	4.35 ± 0.55^b^	2.24 ± 0.00^b^
14:01	0.07 ± 0.18	0.07 ± 0.01	0.25 ± 0.11	0.12 ± 0.09	ND
15:0	0.60 ± 0.19^a^	0.61 ± 0.00^a^	0.36 ± 0.17^ab^	0.12 ± 0.17^b^	0.02 ± 0.00^b^
15:01	0.18 ± 0.20	0.11 ± 0.00	0.11 ± 0.10	ND	ND
16:0	47.29 ± 4.84^a^	34.76 ± 0.44^b^	14.84 ± 1.25^c^	11.68 ± 1.46^c^	10.46 ± 0.00^c^
16:01	1.58 ± 1.88	2.11 ± 0.01	0.56 ± 0.33	0.27 ± 0.21	0.08 ± 0.00
17:0	0.22 ± 0.09	0.22 ± 0.01	0.09 ± 0.02	0.05 ± 0.07	0.09 ± 0.00
17:01	0.11 ± 0.08^ab^	0.19 ± 0.00^a^	0.05 ± 0.06^ab^	0.06 ± 0.08^ab^	ND
18:0	1.95 ± 0.75^c^	4.80 ± 0.10^a^	3.61 ± 0.14^b^	2.76 ± 0.15^b^	5.08 ± 0.00^a^
18:1 (*n*−9)	8.12 ± 2.19^c^	23.93 ± 1.33^b^	45.55 ± 1.59^a^	46.36 ± 1.16^a^	42.00 ± 1.00^a^
18:2	8.92 ± 4.13^c^	17.43 ± 0.17^a^	16.51 ± 1.86^a^	18.72 ± 0.26^a^	18.81 ± 0.00^a^
18:3 n−6	0.58 ± 0.84	0.03 ± 0.00	0.14 ± 0.18	0.08 ± 0.11	ND
18:3 n−3	0.20 ± 0.25^e^	0.88 ± 0.01^d^	2.72 ± 0.29^b^	3.13 ± 0.07^a^	1.73 ± 0.00^c^
20:0	0.56 ± 0.51	0.04 ± 0.00	0.17 ± 0.21	0.34 ± 0.28	0.43 ± 0.00
20:01	0.20 ± 0.10^b^	0.03 ± 0.00^b^	0.47 ± 0.64^a^	ND	ND
20:3 *n*−6	0.22 ± 0.10^a^	0.03 ± 0.00^b^	ND	ND	ND
20:4 *n*−6	0.56 ± 0.16^b^	0.08 ± 0.00^cd^	0.35 ± 0.47^c^	0.86 ± 0.08^a^	ND
20:05	0.19 ± 0.14	0.03 ± 0.00	ND	ND	ND
22:0	0.19 ± 0.14	0.23 ± 0.00	ND	0.12 ± 0.03	ND
20:3*n*−6	0.23 ± 0.13	0.06 ± 0.00	ND	ND	ND
20:3*n*−3	0.10 ± 0.10	0.12 ± 0.16	ND	ND	ND
24:1	0.19 ± 0.11	0.03 ± 0.01	ND	ND	ND
22:6 *n*−3	0.62 ± 0.20^a^	0.60 ± 0.00^b^	ND	ND	ND
SFA	77.75 ± 14.85^a^	54.01 ± 0.35^b^	33.28 ± 2.12^c^	29.70 ± 4.97^c^	37.32 ± 0.13^c^
MUFA	10.46 ± 4.75^c^	26.47 ± 0.34^b^	46.98 ± 3.55^a^	45.64 ± 1.99^a^	42.08 ± 0.00^a^
PUFA	11.61 ± 6.05^b^	19.26 ± 0.18^a^	19.71 ± 1.49^a^	22.60 ± 0.50^a^	20.54 ± 0.02^a^

^a^Values are the mean±SD (n= 3); Values in the same row with different superscript letters (a, b, c, d, e) are significantly different among human milk and infant formulas at (*p* < 0.05).

FA, fatty acid; SFA, saturated fatty acid; MUFA, monounsaturated fatty acid; PUFA, polyunsaturated fatty acid; ND, not detected. Numbers with the same alphabetical letter denote significant differences between these numbers.

Human milk is characterized by high levels of *sn*−*2* SFAs, particularly palmitic acid (16:0), at the *sn–*2 position. These enhance fat absorption and reduce the formation of insoluble calcium soaps in the gut (13). In contrast, IFCM had lower SFA content at the *sn*−*2* position than human milk but higher than that of commercial IFs.

Commercial IFs often use vegetable oils, such as palm oil, which predominantly contain 16:0 esterified at the *sn*−*1* and *sn*−*3* positions. This composition has been associated with increased excretion of FA soap in the stool, firmer stools, and reduced calcium absorption in formula-fed infants compared to exclusively breastfed infants (28, 29). A new approach was developed to redesign IFs by combining CM fat and O/P/O. The newly formulated IFCM has a relatively higher proportion of palmitic acid at the *sn*−*2* position, making it structurally closer to human milk fat. This suggests it can improve fat and calcium absorption compared with commercial IFs (13). However, this newly formulated product was still different from human milk. Further studies are needed to narrow this gap, and physiological studies are required to evaluate the benefits of *sn*−*2* palmitic acid-based IFCM.

The *sn*−*2* positional content of total MUFAs in human milk was 10.46%, two times lower than that of IFCM (26.47%) and four times lower than that of commercial IFs. Notably, the *sn–*2 MUFA levels in IFs were influenced by the type of fat used, with plant-based formulas (commercial IFs) exhibiting much higher *sn–*2 MUFA levels than IFCM.

The level of PUFAs presented at the *sn–*2 position in human milk was 11.61%, slightly lower than all IFs. Other FA differences were found compared to commercial IFs; IFCM has (1) lower 6:0, 8:0, 10:0, and 18:1 (n–9) than commercial IFs, (2) lower 12:0 and 15:0 than IF2 and IF3; and (3) higher 16:0 and SFA than commercial IFs (Table 2). Figures 3B, C shows the PCA, VIP, and heatmap analysis of *sn*−*2* FAs in human milk and all IFs.

The PCA score plot examined the variations in *sn*−*2* FAs in human milk and all IFs (Figure 3B). The first and second principal components account for 95.7% of the variance in the data, which resulted in the division of human milk and all formulas into five groups. This result shows that the overall *sn*−*2* FAs content of the human milk samples differs from that of all IF samples; however, IFCM was closer to the human milk samples. This finding was further supported by VIP and heatmap analysis of *sn*−*2* FA, which contributed most significantly to the separation of all formulas from human milk (Figures 3C, D). PCA and heatmap analyses further supported the similarity between human milk and IFCM, indicating that IFCM was closer to human milk than to commercial IFs.

### 3.4 Lipolysis degree and lipid composition

**Figure 4:**
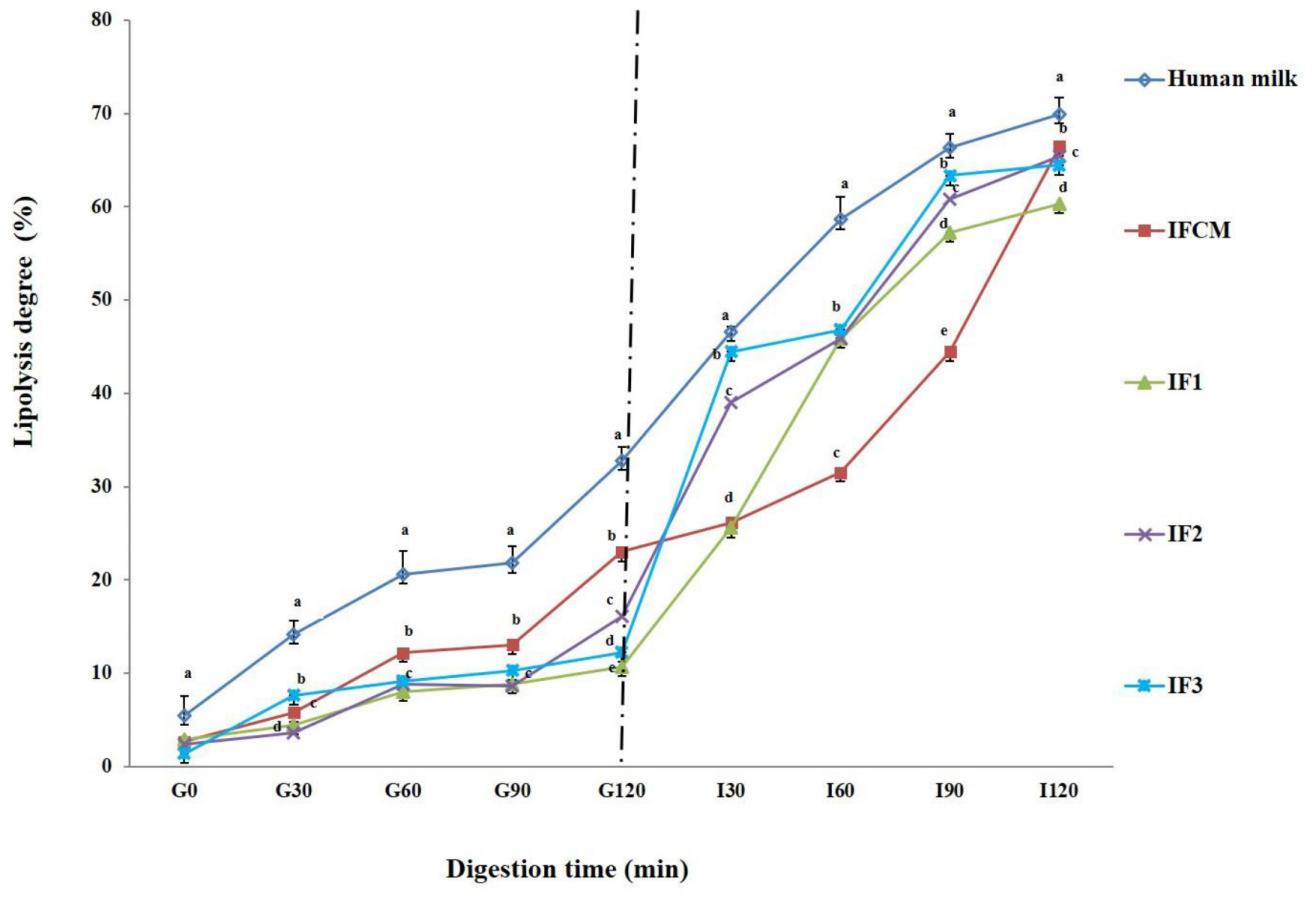
Lipolysis degree of human milk and infant formula during the *in vitro* gastrointestinal digestion. Abbreviations: IF, infant formula; IFCM, infant formula with camel milk. Values are the mean ± SD (*n*= 3); Different letters in the same row indicate significant differences among human milk and IFs (*p* < 0.05).

Digestion of lipids in human milk and IFs involves a complex lipolysis process that releases FAs for subsequent absorption by the body. Therefore, the extent of lipolysis can be used to evaluate changes in lipid hydrolysis products during *in vitro* digestion. Our study examined the degree of lipolysis during the gastric and intestinal phases of digestion for human milk and IFs, as illustrated in Figure 4. Samples were collected from the gastric and intestinal digestion phases at 30, 60, 90, and 120 min. The LD curves of human milk and IFs displayed a remarkably similar pattern. This finding aligns with a previous study that reported a comparable level of LD between human milk and IFs (21). In the gastric phase (G0), human milk demonstrated a higher release level of FFAs than IFs. This pre-lipolysis potentially enhances gastro-digestion by enabling the released FFAs and partial acylglycerols to anchor lipids in the interfacial membrane, facilitating more straightforward fat hydrolysis.

It was observed that during the gastric phase of digestion, lipolysis increased significantly within the first 30 min and then remained almost unchanged from 60 to 90 min. This may be due to the accumulation of FAs during digestion, which further inhibits gastric lipase activity (30) and reduces the substrate concentration available for digestion. Subsequently, from 90 to 120 min, there was a gradual upward trend (Figure 4).

During the gastric phase (G), the LD level in human milk was significantly higher than that in IFs (*P* < 0.05). High LD levels can be attributed to endogenous enzymes in human milk, such as BBSL, and commonly used freezing procedures (31, 32). However, in G120, the LD level of the IFCM was higher than that of commercial IFs. This similarity in digestion suggests that IFCM is more closely aligned with the composition of human milk. Chai et al. (33) reported that IFs prepared from milk fat globule membranes lack the natural fat globule structure and have a thicker interfacial layer, reducing lipase action compared to human milk fat globules. As a result, the fat globule component in CM is believed to have a comparable role to the fat globule component in human milk.

Notably, the LDs in IFCM demonstrated a slower rate of increase before 90 min during intestinal digestion (Figure 4). Several factors may contribute to this phenomenon. First, our previous findings indicate that CM fat globules contain a significantly higher PL content than human milk (12). A phospholipid-coated interfacial structure in the lipid droplets of IFs hinders their reactivity in the intestinal phase, thereby limiting the entry of digestive lipase into the TAG core (34). Additionally, after gastric digestion, the protein matrix and emulsion stability in IFCM may further impede pancreatic lipase's early action, thereby delaying lipolysis. This is because the protein matrix can form an interpolymeric gel network around the emulsion droplets, hindering pancreatic lipase's access tohe lipid droplets (35). The presence of a protein matrix can also limit the emulsification property of hydrolysis products, leading to a lower contact area between the lipids and pancreatic lipase (36). Furthermore, various surface-active peptides are generated during gastric digestion, differing in their effects on the stabilization and breakdown of fat globules (37). However, further in-depth studies of gastric and intestinal transit of IFCM are needed to understand the behavior of non-CM fat in infants.

**Figure 5:**
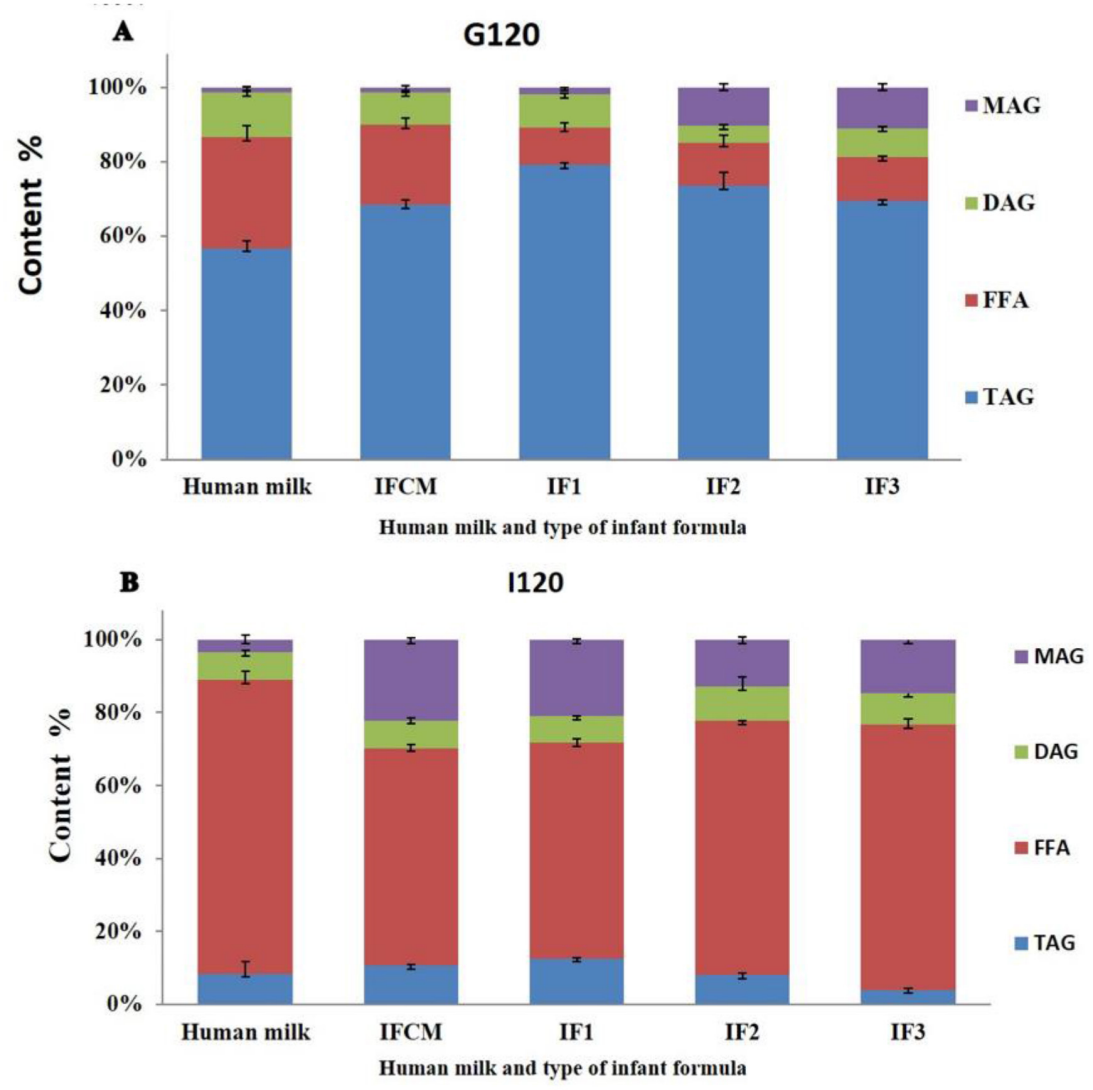
Content of triacylglycerol (TAG), diacylglycerol (DAG), monoacylglycerol (MAG), and free fatty acids (FFAs) in human milk and infant formulas G120 **(A)**, I120 **(B)**. IF, infant formula; IFCM, infant formula with camel milk; G, gastric-Phase; I, intestinal-phase.

At G120, the concentration of TAGs in human milk was significantly lower than that in all formulas (*P* < 0.05). On the other hand, the levels of FFAs (*P* < 0.05) and DAGs (*P* < 0.05) were noticeably higher, thereby illustrating the rapid digestion of human milk; however, IFCM was closer to human milk fat (Figure 5). Upon entering the intestinal phase, both human milk and IFs exhibited a significant increase in LD (*P* < 0.05). The LDs displayed a gradual upward trend as the digestion process progressed, although the growth rate decelerated after 90 min. At I120, the level of LDs followed the order: IF1 < IF3 < IF2 < IFCM < human milk, with respective amounts of 60.28 ± 0.51%, 64.45 ± 0.61%, 65.43 ± 0.31%, 66.47 ± 0.21%, and 69.93 ± 1.90%. This ordering indicates that the PL protective layer of fat globules in IFCM gradually erodes, exposing more TAGs for improved interaction with pancreatic lipase. Slower initial lipolysis in IFCM may have nutritional implications. These *in vitro* findings suggest a different lipid digestion pattern, which may warrant further investigation *in vivo*.

### 3.5 Comparison of free fatty acids released during gastrointestinal digestion of human milk and infant formulas

**Figure 6:**
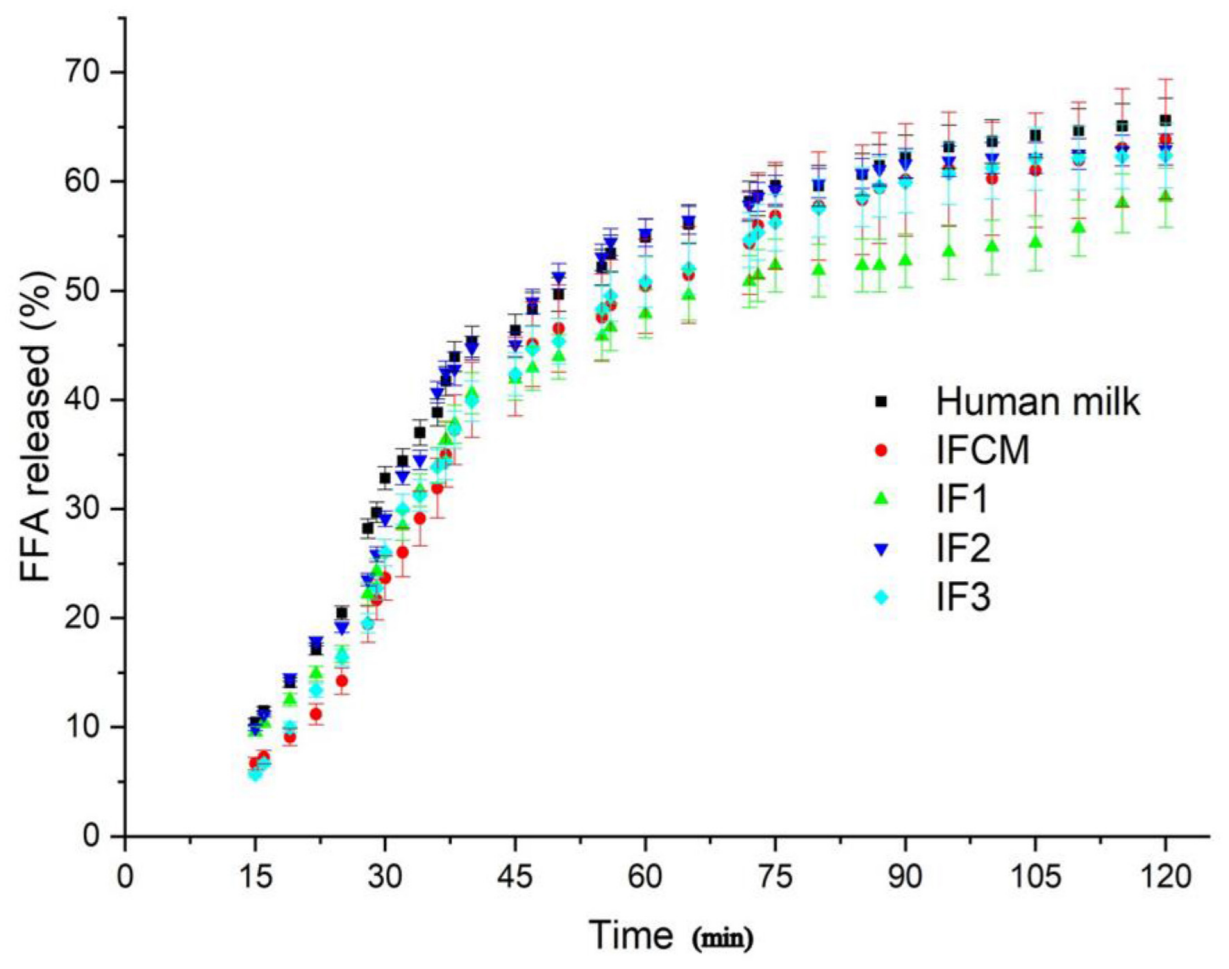
FFA release of human milk and infant formulas during the *in vitro* gastrointestinal digestion.

Measuring FFA release using pH-stat monitoring can assess the rate of FFA production during intestinal digestion. Figure 6 illustrates the FFA released from human milk and all formulas. The release of FFAs showed an upward trend in all samples, starting at 15 min and continuing until the end of digestion. No data were available before the 15 min mark because the liquid level was below the pH meter during the initial 15 min of the dynamic model.

During the early stages of digestion (first 25 min), human milk and commercial IFs released more FFAs than IFCM, although this difference was not statistically significant. However, this difference did not reach statistical significance. This early lipolytic activity is mainly due to the FA composition of the formulas. As shown in Table 1, human milk and commercial IFs contain a greater proportion of MCFAs, which are known to be more readily hydrolyzed by pancreatic lipase due to their shorter chain length and higher solubility (38, 39).

Interestingly, as digestion progressed beyond the initial phase, IFCM gradually increased FFA release, eventually surpassing most commercial IFs. This delayed but sustained lipolytic response may be linked to the unique FA profile of CM, which contains a higher proportion of long-chain FAs and a distinctive milk fat globule membrane composition (40, 41). The complex structure of the milk fat globule membrane and the predominance of long-chain FAs could slow initial lipase action but promote more efficient fat hydrolysis over extended digestion periods (42). The gradual increase in FFA release from IFCM suggests that its lipid structure and composition may support a more prolonged lipid digestion, potentially providing sustained energy release and improved bioavailability of essential FAs (43). However, potential effects on infant growth, mineral absorption, gut health, or metabolism remain hypothetical and require confirmation in clinical studies.

The rate of FFA release varied among samples, with some exhibiting rapid increases within the first 60 min and others showing slower release between 60 and 120 min. These findings align with a pH-stat-monitored study on human milk fat digestion (22). This demonstrated an initial rapid release of FFA over the first 17 min, followed by a gradual rise to a relatively stable plateau. Subsequently, the release of FFAs from the IFs showed a slower increase from 30 to 50 min, and the FFA release level of IFCM gradually exceeded that of most commercial IFs. These compositional and structural differences underscore the significance of milk fat source in influencing the digestibility and kinetics of nutrient release from IF.

### 3.6 Fourier transform infrared of human milk and infant formula profiles

**Figure 7:**
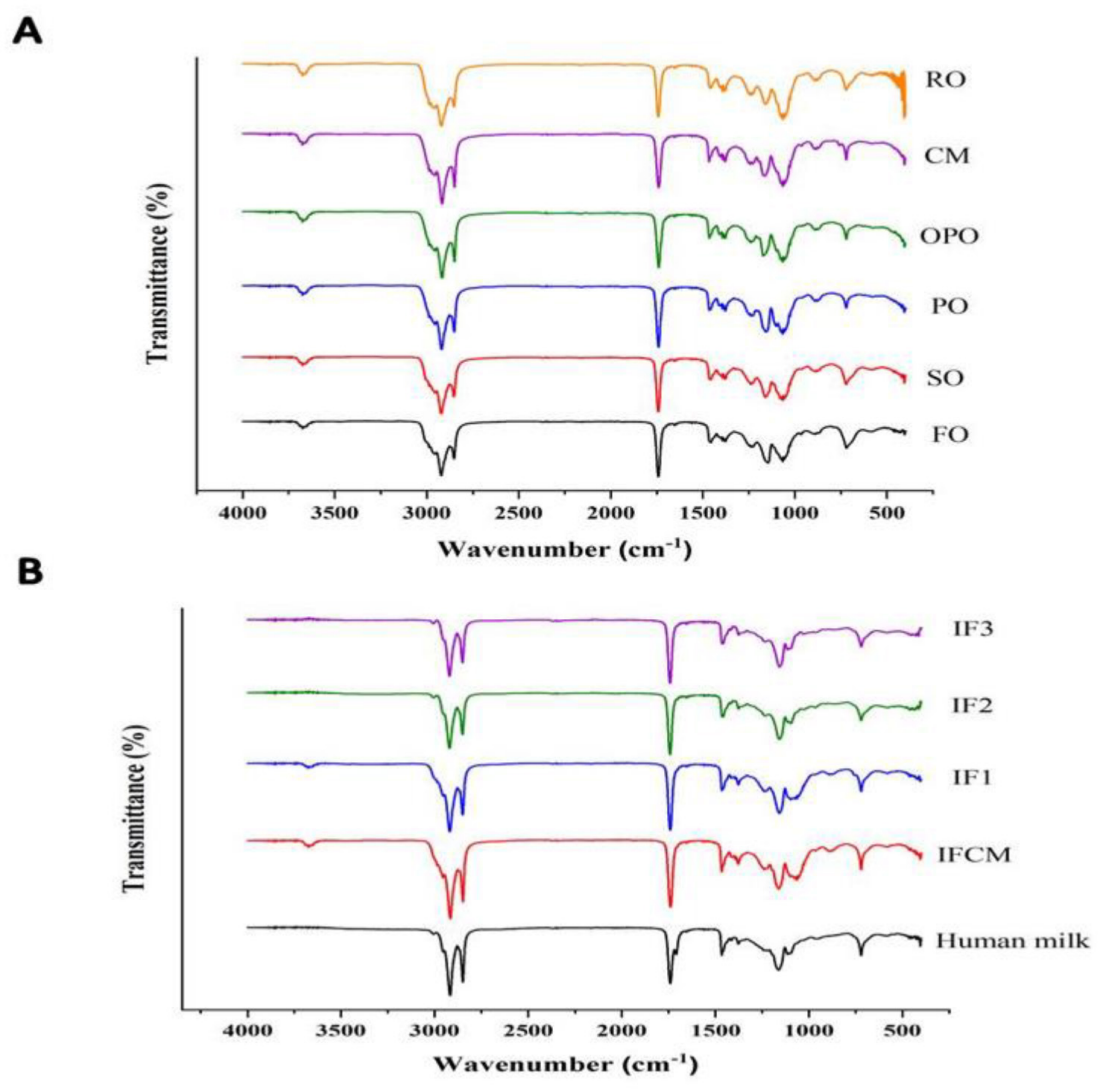
The infrared spectrum for camel milk fat with selected oils **(A)**, human milk, and infant formulas **(B)**. IF, infant formula; IFCM, infant formula with camel milk; SO, sunflower oil; FO, fish oil; RO, rapeseed oil; O/P/O, 1,3-dioleoyl-2-palmitoylglycerol; PO, palm kernel oil.

FTIR is highly effective at providing comprehensive information on the composition and properties of milk fats. This is primarily due to the distinctive absorption peak corresponding to carbonyl bonds, which typically appear within the range of 1,800 to 1,700 cm^−1^, offering a characteristic pattern for total fats. Moreover, the absorption peaks within the 3,050–3,000 cm^−1^ range correspond to the olefinic double bonds (–HC=CH–) found in unsaturated FA. Figure 7A displays the characteristic peaks obtained through ATR-FTIR in the mid-infrared region (4,000–400 cm^−1^) for CM's fat/oil fractions and various types of oils. The spectral patterns of different fats/oils showed similarities, such as the absorption bands of edible oils (44). Similarly, a comparative analysis of the spectral patterns of human milk and IF samples is presented in the Figure 7B. The major absorption bands of human milk and all IF samples were similar, with some differences in the individual absorption bands. For example, human milk, IF2, and IF3 exhibited a weak absorption band at 3,008.08 cm^−1^, associated with the stretching vibration of the –C=C–H in cis-unsaturated bonds. However, this absorption band was not observed compared to the IFCM and IF1 (Figure 7B). Several factors may contribute to this phenomenon; this is likely due to spectral overlap with adjacent –CH_2_ and –CH3 stretching bands (2,850–2,950 cm^−1^), causing the peak to merge into a broader absorption rather than disappear entirely (45). Additionally, the relatively lower unsaturation levels in IFCM and IF1 compared to other samples may reduce the intensity of this band, making it less prominent and more easily masked (41). Variations in fat globule size, matrix composition, and interactions with proteins can also influence peak shape and resolution, contributing to the merging effect (46). Therefore, the apparent absence of a distinct 3,008 cm^−1^ absorption does not indicate a lack of unsaturated fatty acids but reflects the complex spectral characteristics of lipid mixtures in these formulas.

Furthermore, the differences between human milk and all IFs were observed at 1,707 cm^−1^. Human milk displayed absorption peaks at 1,707 cm^−1^, while the IFs did not exhibit the same absorption peaks. The observed differences in the spectral regions between human milk and IF can be attributed to variations in the composition of their respective fat fractions, particularly in chain length and degree of unsaturation.

Nevertheless, the most prominent bands are typically associated with C-H stretching (2,850–3,000 cm^−1^), C=O stretching (1,700–1,750 cm^−1^), C-H deformation (1,200–1,470 cm^−1^, 720 cm^−1^), and C-O stretching (1,160 cm^−1^) vibrations (47). In addition, the bands observed at 1,710 cm^−1^ and 1,570 cm^−1^ have been linked explicitly to unrestricted FA (48). These distinctive absorption bands provide insights into the presence and composition of various functional groups within the analyzed samples.

### 3.7 Crystallization and melting of human milk and infant formula profiles

**Figure 8:**
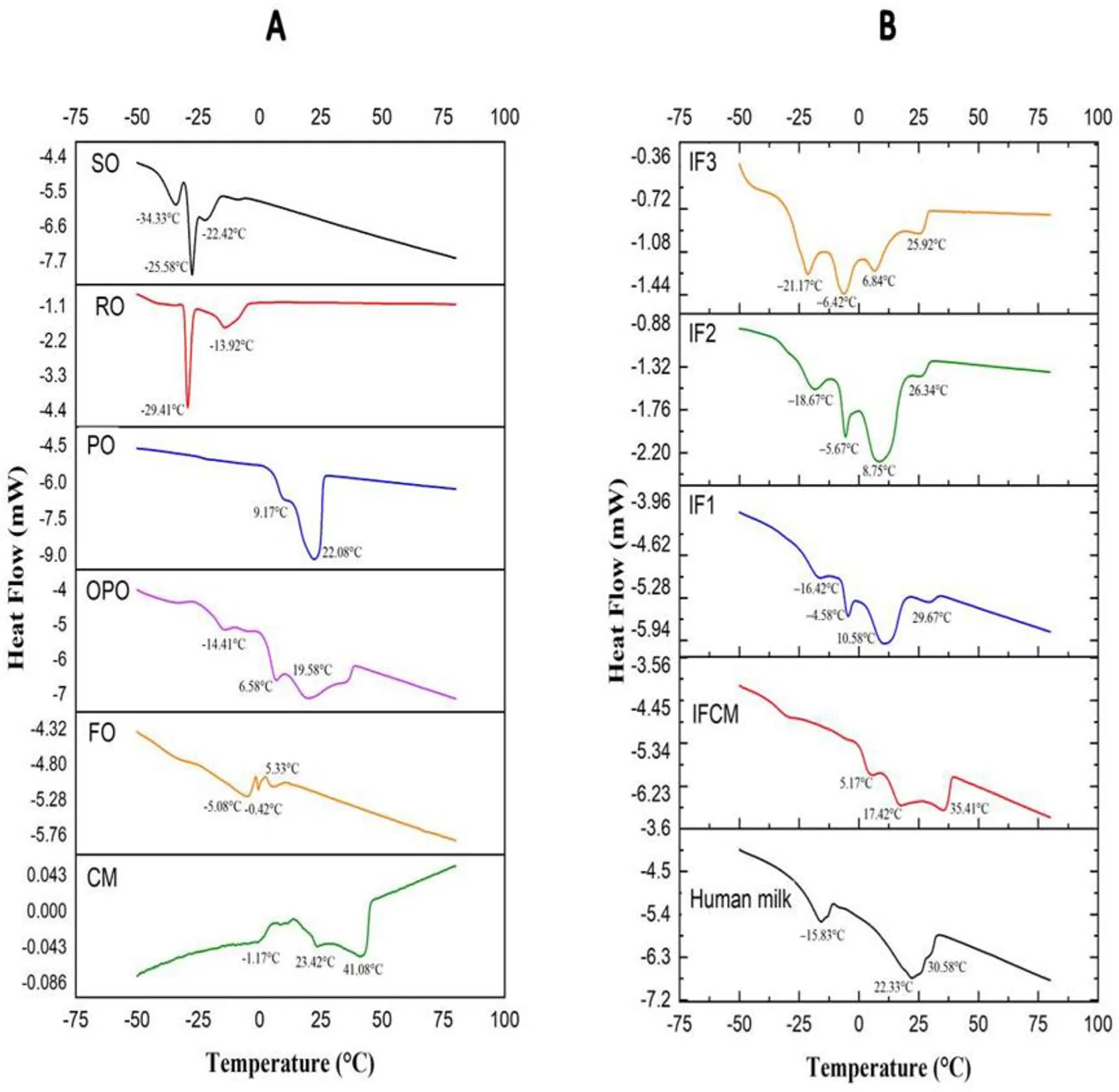
DSC curves of melting profiles for camel milk fat with selected oils **(A)**, human milk, and infant formula **(B)**. IF, infant formula; IFCM, infant formula with camel milk; SO, sunflower oil; FO, fish oil; RO, rapeseed oil; O/P/O, 1,3-dioleoyl-2-palmitoylglycerol; PO, palm kernel oil.

During IF development, optimizing the FA composition and triacylglycerol distribution to support lipid digestion is crucial. Therefore, the melting and crystallization properties of CM fat and selected oils were examined (Figure 8). The individual ingredients showed distinct thermal behaviors, with CM fat displaying a major melting transition at 41.08 °C, while SO, FO, OPO, RO, and PO exhibited lower or broader transitions across subambient to near-physiological temperatures. After blending, IFCM showed a modified thermal profile compared to CM fat, indicating that the formulation changed its crystallization and melting characteristics. Consequently, the final melting temperatures of fat in human milk and all infant formulas were below the physiological temperature range of 36.6–37.3 °C, which facilitates rapid absorption (Figure 8B).

Human milk showed lower final melting temperatures than IFCM, while the commercial formulas showed even lower melting temperatures than human milk fat. This difference is attributed to the composition of fats: human milk and IFCM contain long-chain SFAs with higher melting points, whereas commercial IFs contain more short- and medium-chain SFAs with lower melting points (49). The melting properties of fats affect the formation and stability of micelles, which are crucial for fat absorption. Fats with lower melting temperatures readily form micelles, facilitating the absorption of FA and monoglycerides. In contrast, fats with higher melting temperatures may form fewer stable micelles or require more energy to form, potentially impacting absorption efficiency (49).

**Figure 9:**
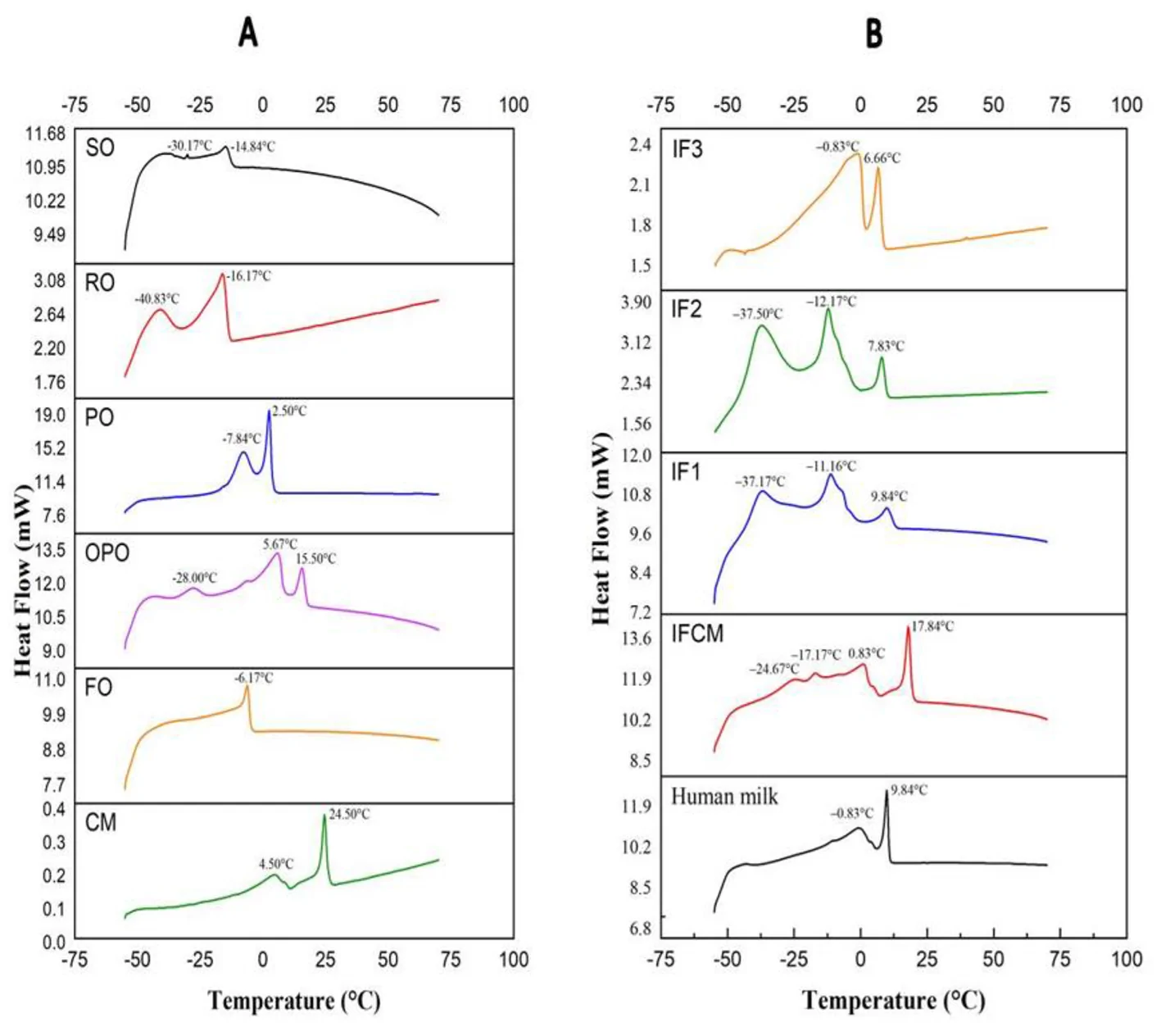
DSC curves of crystallization profiles for camel milk fat with selected oils **(A)**, human milk, and infant formulas **(B)**. IF, infant formula; IFCM, infant formula with camel milk; SO, sunflower oil; FO, fish oil; RO, rapeseed oil; O/P/O, 1,3-dioleoyl-2-palmitoylglycerol; PO, palm kernel oil.

Crystallization curve revealed crystal peaks at 24.50, −14.84, −6.17, 15.50, −16.17, and 2.50 °C for CM fat, SO, FO, O/P/O, RO, and PO, respectively (Figure 9A). Although the crystallization properties of fat in IF may not directly affect digestion, they tend to influence its functionality and storage behavior in the formulation of IF products (50). According to the crystallization curves (Figure 9B), it is observed that higher starting crystallization temperatures of IF fats correspond to higher final melting temperatures, and vice versa. IFCM fat had the highest starting crystallization temperature (23.1 °C), corresponding to its highest final melting temperature (42.4 °C) (51), indicating a more stable crystalline structure than other IFs.

The present findings suggest that CM fat, when combined with selected vegetable and marine oils, can be used to design a lipid system that more closely resembles selected compositional and structural features of human milk fat than conventional plant oil-based formulas. From a biological perspective, the closer match in total and *sn-2* FA distribution may be relevant because these characteristics can influence lipid hydrolysis behavior and the physical form of digestion products. However, the current data are limited to compositional, thermal, and *in vitro* digestion analyses, and therefore do not permit conclusions about infant growth, mineral absorption, or broader metabolic outcomes. Future *in vivo* studies will be needed to determine whether the observed structural similarities translate into meaningful nutritional advantages.

## 4 Conclusion

A novel IFCM was designed to closely replicate the FA composition and digestion of human milk. CM fat was optimized by blending it with sunflower, rapeseed, palm kernel, fish, and 1,3-dioleoyl-2-palmitoylglycerol oils using a Python-based computational model to achieve a composition resembling that of human milk. Comparative analyses with human milk and three commercial formulas assessed total and *sn–*2 positional FA profiles, physicochemical properties, and *in vitro* digestion characteristics. IFCM demonstrated a higher proportion of palmitic acid at the *sn*−*2* position (34.76%) and exhibited fewer compositional deviations from human milk than commercial formulas. The intestinal lipolysis degree of IFCM (66.47%) was comparable to human milk (69.93%), indicating improved fat hydrolysis efficiency. Fourier transform infrared and thermal analyses confirmed substantial structural similarity between IFCM and human milk fats. These results reveal that combining CM fat with selected oils enhances the nutritional and functional properties of infant formulas, improving digestibility and potentially conferring physiological benefits. This formulation offers a promising framework for developing next-generation infant nutrition products with greater biofunctional equivalence to human milk. Future research should focus on assessing the *in vivo* physiological effects of IFCM on infant growth, development, and gut health.

## Data Availability

The original contributions presented in the study are included in the article/Supplementary material, further inquiries can be directed to the corresponding authors.
