# Supplementary material for: Camel milk fat-based human milk fat substitute
Source: Front Nutr. 2026 Sep 4;13:1917713. doi: 10.3389/fnut.2026.1917713 (PMC13587610; doi:10.3389/fnut.2026.1917713)
Supplement: Supplementary file 1 [file Table_1.docx]

Supplementary Table 1. Characteristics of the camels and their corresponding milk samples

| Variables | Camels |
| --- | --- |
| Number of samples | 20 |
| Age (years) | 5.5 ± 0.17 |
| Quantity of milk/ week (L) | 35 |
| Season | Winter |
| Type of Food | Barley, corn, wheat, and mineral blocks |
| Time of Sampling | Early morning |
| Water (%) | 87.53± 0.07 |
| Total solids (%) | 12.44± 0.08 |
| Fat (%) | 4.20± 0.05 |
| Protein (%) | 3.33± 0.04 |
| Ash (%) | 0.73± 0.14 |
| Lactose (%) | 4.34± 0.07 |
| PH | 6.6± 0.02 |

Supplementary Table 2. The total fatty acid composition of camel milk and various oils.

| FAs | Fish oil | Sunflower oil | Rapeseed oil | O/P/O | Palm kernel oil | Camel milk |
| --- | --- | --- | --- | --- | --- | --- |
| 6:0 | ND | ND | 0.03±0.01 | ND | 0.20±0.00 | 0.13±0.00 |
| 8:0 | ND | ND | ND | 0.02±0.00 | 3.16±0.01 | 0.21±0.00 |
| 10:0 | ND | ND | ND | 0.04±0.00 | 3.06±0.01 | 0.23±0.00 |
| 11:0 | ND | ND | ND | ND | 0.06±0.00 | ND |
| 12:0 | 0.13±0.00 | ND | 0.02±0.01 | 0.21±0.01 | 41.01±0.00 | 1.05±0.01 |
| 13:0 | 0.06±0.00 | ND | ND | ND | 0.05±0.00 | 0.09±0.01 |
| 14:0 | 7.21±0.01 | ND | 0.10±0.00 | 0.62±0.01 | 13.92±0.03 | 15.53±0.06 |
| 14:01 | 0.25±0.03 | ND | 0.00±0.00 | ND | ND | 0.46±0.64 |
| 15:0 | 0.74±0.01 | ND | 0.07±0.01 | 0.04±0.00 | ND | 1.52±0.00 |
| 15:01 | 0.14±0.02 | ND | ND | ND | ND | 0.22±0.24 |
| 16:0 | 19.34±0.06 | 6.63±0.06 | 15.67±0.08 | 35.86±0.18 | 10.58±0.04 | 30.97±0.16 |
| 16:01 | 8.60±0.00 | 0.09±0.01 | 0.17±0.00 | 0.08±0.00 | 0.02±0.00 | 7.51±0.05 |
| 17:0 | 0.80±0.06 | 0.03±0.00 | 0.05±0.00 | 0.09±0.01 | ND | 0.86±0.00 |
| 17:01 | 1.00±0.01 | 0.03±0.01 | 0.04±0.00 | ND | ND | 0.30±0.42 |
| 18:0 | 3.56±0.01 | 3.54±0.08 | 1.00±0.02 | 4.02±0.05 | 2.82±0.02 | 15.35±0.18 |
| 18:1 (n-9) | 15.43±0.10 | 24.88±0.07 | 15.13±0.04 | 50.71±0.18 | 21.39±0.01 | 21.22±1.27 |
| t18:2 | 3.14±0.00 | 0.64±0.06 | 1.16±0.01 | ND | 3.60±0.00 | 0.68±0.06 |
| 18:2 | 3.91±0.05 | 62.95±0.07 | 58.03±0.03 | 7.51±0.04 | ND | 2.48±0.07 |
| 18:3n-6 | 0.30±0.05 | ND | 0.01±0.01 | ND | ND | 0.13±0.01 |
| 18:3n-3 | 1.13±0.01 | 0.09±0.01 | 6.30±0.04 | 0.22±0.01 | ND | 0.46±0.01 |
| 20:0 | 0.61±0.01 | 0.18±0.01 | 0.15±0.00 | 0.27±0.01 | ND | 0.45±0.05 |
| 20:01 | 2.66±0.04 | 0.14±0.06 | 1.23±0.01 | 0.19±0.00 | ND | 0.05±0.06 |
| 20:2n-6 | ND | ND | 0.13±0.00 | ND | ND | ND |
| 22:0 | 1.06±0.20 | 0.76±0.01 | 0.09±0.00 | 0.16±0.00 | ND | 0.06±0.08 |
| 22:01 | 3.54±0.17 | ND | 0.34±0.04 | ND | ND | ND |
| 20:3n-6 | ND | ND | ND | ND | ND | ND |
| 20:3n-3 | ND | ND | ND | ND | ND | 0.07±0.09 |
| 20:4n-6 | 1.00±0.04 | ND | 0.01±0.00 | ND | ND | ND |
| 20:05 | 12.13±0.01 | ND | ND | ND | ND | ND |
| 24:01 | 0.89±0.07 | ND | 0.10±0.01 | ND | ND | ND |
| 22:6 n-3 | 12.39±0.01 | ND | 0.15±0.03 | ND | ND | 0.05±0.06 |
| MC-SFA | 0.13±0.00 | ND | 0.05±0.00 | 0.26±0.07 | 47.43±0.00 | 1.61±0.00 |
| LC-SFA | 32.56±0.11 | 11.31±0.16 | 18.38±0.09 | 40.72±0.16 | 27.36±0.01 | 63.95±0.18 |
| SAF | 33.49±0.00 | 11.34±0.16 | 18.48±0.09 | 41.07±0.16 | 74.79±0.01 | 66.43±0.19 |
| MUFA | 31.58±0.12 | 25.10±0.03 | 15.47±0.03 | 50.79±0.13 | 21.41±0.01 | 29.28±0.01 |
| PUFA | 21.84±0.09 | 63.58±0.10 | 65.76±0.27 | 8.15±0.03 | 3.74±0.05 | 3.85±0.21 |
| LA/ALA | 3.47±0.01 | 698.33±1.11 | 386.86±1.06 | 28.34±0.64 | ND | 5.45±0.05 |

Values are the mean ± SD (*n* = 3); Abbreviations are: FAs, fatty acids; SFA, saturated fatty acids; MC-SFA, medium-chain SFA; LC-SFA, long chain; MUFA, monounsaturated fatty acids; PUFA, polyunsaturated fatty acids; LA/ALA, linoleic acid/alpha-linolenic acid; ND, not detected.

Supplementary Table 3. The *sn-2* fatty acid composition of camel milk and various oils.

| FA | Fish oil | Sunflower oil | Rapeseed oil | O/P/O | Palm kernel oil | Camel milk |
| --- | --- | --- | --- | --- | --- | --- |
| 4:0 | 0.31±0.01 | 0.19±0.02 | 0.12±0.02 | ND | ND | 0.21±0.01 |
| 6:0 | 0.54±0.01 | 0.32±0.03 | 0.16±0.00 | 0.02±0.00 | 0.13±0.00 | 0.24±0.06 |
| 08:0 | 0.75±0.00 | 0.61±0.01 | 0.27±0.04 | ND | 0.16±0.04 | 0.37±0.02 |
| 10:0 | 0.72±0.02 | 0.36±0.00 | ND | ND | 0.87±0.03 | ND |
| 12:0 | 0.09±0.01 | ND | 0.04±0.01 | 0.25±0.01 | 38.32±0.01 | 0.55±0.02 |
| 14:0 | 11.11±0.07 | 0.05±0.02 | 0.03±0.02 | 0.90±0.02 | 11.68±0.01 | 15.2±0.07 |
| 14:01 | 0.50±0.00 | ND | 0.02±0.00 | ND | ND | 0.59±0.01 |
| 15:0 | 0.94±0.05 | ND | ND | ND | ND | 1.83±0.07 |
| 15:01 | 0.15±0.00 | ND | ND | ND | ND | 0.47±0.03 |
| 16:0 | 26.83±0.04 | 4.15±0.06 | 3.48±0.02 | 66.17±0.21 | 5.71±0.04 | 40.46±0.07 |
| 16:01 | 7.95±0.06 | 0.1±0.07 | 0.13±0.01 | ND | ND | 7.60±0.07 |
| 17:0 | 0.46±0.05 | ND | ND | ND | ND | 0.60±0.00 |
| 17:01 | 0.97±0.03 | ND | ND | ND | ND | 0.4±0.00 |
| 18:0 | 10.47±0.02 | 6.32±0.02 | 3.80±0.00 | 6.24±0.03 | 1.45±0.01 | 8.74±0.06 |
| 18:1 (n-9) | 9.38±0.01 | 21.89±0.07 | 15.24±0.04 | 21.46±0.05 | 35.28±0.04 | 19.14±0.03 |
| t18:2 | 0.99±0.00 | ND | ND | ND | ND | 0.3±0.07 |
| 18:2 | 5.26±0.03 | 65.39±0.01 | 70.96±0.03 | 3.50±0.01 | 5.50±0.00 | 2.11±0.07 |
| 18:3n-6 | 0.17±0.02 | ND | ND | ND | ND | 0.05±0.00 |
| 18:3n-3 | 0.78±0.02 | 0.05±0.01 | 5.16±0.03 | ND | ND | 0.12±0.02 |
| 20:0 | 0.19±0.01 | 0.12±0.03 | 0.06±0.02 | ND | 0.05±0.07 | 0.40±0.14 |
| 20:01 | 0.63±0.04 | 0.07±0.00 | 0.07±0.02 | 0.21±0.01 | ND | 0.40±0.14 |
| 20:2n-6 | 0.16±0.00. | ND | ND | ND | ND | 0.07±0.02 |
| 22:0 | 0.89±0.01 | 0.14±0.04 | 0.09±0.01 | 0.11±0.00 | ND | 0.06±0.03 |
| 20:3n-6 | 0.70±0.00 | ND | ND | ND | 0.69±0.01 | ND |
| 20:3n-3 | 1.64±0.01 | ND | ND | ND | ND | 0.33±0.04 |
| 20:4n-6 | 0.56±0.04 | 0.18±0.01 | 0.15±0.02 | 0.30±0.00 | ND | ND |
| 20:05 | 5.60±0.00 | ND | 0.12±0.04 | ND | ND | 0.07±0.04 |
| 24:01 | 0.59±0.01 | ND | ND | ND | ND | 0.01±0.00 |
| 22:6 n-3 | 11.65±0.08 | 0.07±0.01 | 0.09±0.02 | ND | ND | 0.01±0.00 |
| SFA | 53.26±0.02 | 12.24±0.06 | 8.02±0.08 | 74.10±0.17 | 58.39±0.04 | 68.65±0.06 |
| MUFA | 20.16±0.06 | 22.06±0.00 | 15.45±0.06 | 21.62±0.01 | 35.28±0.04 | 28.61±0.01 |
| PUFA | 26.50±0.10 | 65.69±0.02 | 76.47±0.08 | 3.80±0.21 | 6.24±0.00 | 2.75±0.06 |

Values are the mean±SD (*n* = 3); Abbreviations: FAs, fatty acids; SFA, saturated fatty acids; MUFA, monounsaturated fatty acids; PUFA, polyunsaturated fatty acids; ND, not detected.

Supplementary Table 4. Theoretical and actual values of the final product.

| FA | Theoretical values | | Actual values | |
| --- | --- | --- | --- | --- |
|  | Total | *sn-2* | Total | *sn-2* |
| 6:0 | 0.09 | 0.19 | 0.15 | 0.02 |
| 8:0 | 0.56 | 0.28 | 0.45 | 0.01 |
| 10:0 | 0.56 | 0.19 | 0.48 | 0.11 |
| 12:0 | 6.62 | 6.06 | 6.09 | 5.95 |
| 13:0 | 0.09 | 0.05 | 0.04 | 0.06 |
| 14:0 | 8.80 | 8.59 | 7.33 | 7.19 |
| 14:01 | 0.19 | 0.26 | 0.19 | 0.07 |
| 15:0 | 0.66 | 0.76 | 0.60 | 0.61 |
| 15:01 | 0.09 | 0.19 | 0.13 | 0.11 |
| 16:0 | 24.81 | 33.24 | 24.65 | 34.76 |
| 16:01 | 3.48 | 2.64 | 2.27 | 2.11 |
| 17:0 | 0.41 | 0.28 | 0.34 | 0.22 |
| 17:01 | 0.18 | 0.21 | 0.17 | 0.19 |
| 18:0 | 7.89 | 6.39 | 10.40 | 4.80 |
| 18:1 (*n*-9) | 26.09 | 21.07 | 27.87 | 23.93 |
| t18:2 | 1.18 | ND | 0.20 | ND |
| 18:2 | 14.53 | 16.52 | 15.62 | 17.43 |
| 18:3 n-6 | 0.07 | 0.03 | 0.05 | 0.03 |
| 18:3 n-3 | 1.23 | 0.87 | 1.14 | 0.88 |
| 20:0 | 0.29 | 0.23 | 0.34 | 0.04 |
| 20:01 | 0.38 | 0.29 | 0.14 | 0.03 |
| 20:2 n-6 | 0.02 | 0.03 | 0.01 | 0.03 |
| 20:4 n-6 | 0.24 | 0.56 | 0.24 | 0.08 |
| 20:3 n-3 | 0.03 | 0.33 | 0.05 | 0.18 |
| 20:05 | 0.51 | 0.31 | 0.44 | 0.03 |
| 22:0 | 0.16 | 0.10 | 0.15 | 0.23 |
| 22:01 | 0.23 | ND | 0.07 | ND |
| 24:01 | 0.05 | 0.03 | 0.04 | 0.03 |
| 22:6 n-3 | 0.56 | 0.60 | 0.46 | 0.60 |
| SAF | 50.85 | 56.39 | 50.99 | 54.01 |
| MUFA | 30.69 | 24.68 | 30.80 | 26.47 |
| PUFA | 18.31 | 18.83 | 18.20 | 19.26 |

Abbreviations: FA, fatty acid; SFA, saturated fatty acid; MUFA, monounsaturated fatty acid; PUFA, polyunsaturated fatty acid; ND, not detected.
